# Tuning the Mechanical and Electrical Properties of Porous Electrodes for Architecting 3D Microsupercapacitors with Batteries‐Level Energy

**DOI:** 10.1002/advs.202004957

**Published:** 2021-06-20

**Authors:** Congming Li, Xiangming Li, Qingzhen Yang, Pengcheng Sun, Lifeng Wu, Bangbang Nie, Hongmiao Tian, Yingche Wang, Chunhui Wang, Xiaoliang Chen, Jinyou Shao

**Affiliations:** ^1^ Micro‐/Nano‐technology Research Center State Key Laboratory for Manufacturing Systems Engineering Xi'an Jiaotong University Xi'an Shaanxi 710049 China; ^2^ State Key Laboratory of High Performance Complex Manufacturing Central South University Changsha Hunan 410000 China; ^3^ The Key Laboratory of Biomedical Information Engineering of Ministry of Education Bioinspired Engineering and Biomechanics Center (BEBC) School of Life Science and Technology Xi'an Jiaotong University Xi'an Shaanxi 710049 China; ^4^ Department of Materials Science and Engineering Materials Research Laboratory University of Illinois at Urbana‐Champaign Urbana Illinois 61801 USA; ^5^ Xi'an Institute of Electromechanical Information Technology Xi'an Shaanxi 710065 China

**Keywords:** 3D electrodes, electrical‐mechanical performance, gel electrolytes, microsupercapacitors, solid‐state supercapacitors

## Abstract

Microsupercapacitors (MSCs) are vital power sources for internet of things (IoTs) and miniaturized electronics. The performance of MSCs is often restricted by its low areal energy density, which is due to the low areal mass loading of active materials. Constructing thick planar microelectrode with fine structure and high aspect ratio is an efficient way to increase mass loading, but limited by the breakable nature of porous electrode materials. Here, it is found that the mechanical and electrical properties of porous electrodes, as well as their surface area utilization and internal ion diffusion pathway, can be synergistically tuned by infilling gel electrolyte into internal pores of porous electrode films. The tuned thick porous electrode films are robust enough to enable laser ablation of three dimensional (3D) microelectrodes for high mass loading and high aspect ratio. The areal capacitance of 3D microelectrodes is able to increase linearly with mass loading (or thickness) up to at least 13 mg cm^−2^ (or 260 µm) for a value of up to 4640 mF cm^−2^ based on active carbon. The 3D MSCs deliver areal energy density of 1318 μWh cm^−2^, which is comparable to the best of Li‐ion 3D microbatteries while exhibiting superior electrochemical and mechanical stability.

## Graphical abstract

The mechanical and electrical properties of thick electrode films are tuned by infilling gel electrolyte, enabling laser ablation of 3D microsupercapacitors (MSCs) with high mass loading and high aspect ratio. The MSCs deliver an energy density of 1318 μWh cm^−2^, comparable to the best of microbatteries while exhibiting a superior power density of 102 mW cm^−2^ with high mechanical and cycling stability.

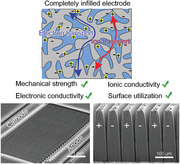

## 1 Introduction

Miniaturized electrochemical energy storage devices are essential for the building blocks of internet of things (IoTs), such as microelectronics, distributed sensors, and wireless communication transmitters. The miniaturized electric double‐layer capacitors (EDLCs), also known as microsupercapacitors (MSCs), store charges at the interface of the electrode materials and electrolytes, exhibiting advantages of high power density, super long cycle life, and high reliability compared with Li‐ion microbatteries or thin‐film batteries.^[^ ^1^, ^2^, ^3^, ^4^, ^5^ ^]^ However, MSCs typically have low areal energy density, often less than tens of microwatt hour per square centimeter, at least one order of magnitude lower than that of Li‐ion micro‐batteries,^[^ ^2^, ^3^, ^4^, ^5^, ^6^, ^7^, ^8^, ^9^, ^10^, ^11^, ^12^, ^13^, ^14^, ^15^ ^]^ limiting their widespread use.

The areal energy density of MSCs is proportional to areal capacitance (*C*_A_), which is dominated by the mass loading (*M*_A_, or thickness, *H*) of the microelectrodes and gravimetric specific capacitance (*C*_m_, or volumetric specific capacitance, *C*_v_) of the used electrode materials. As shown in formula (1),

(1) $$C_{A}=\frac{1}{4}M_{A}C_{m},\mathrm{or}\frac{1}{4}\frac{k}{k+1}HC{}_{V}$$

where *k* refers to the duty factor for microelectrode finger width to the interspace. The electrode materials of supercapacitors have been well developed in the past decades, exhibiting volumetric capacitance of up to hundreds of farads per cubic centimeter for electric double‐layer (EDL) materials, or gravimetric capacitance of more than one thousands of farads per gram for pseudocapacitive materials. However, one of main drawback in realization of high areal energy MSCs is the limited areal capacitance due to low mass loading per unit area on the substrate. The mass loading of in‐plane microelectrodes is typically within 1 mg cm^−2^, corresponding to thickness of submicron to several microns,^[^ ^2^, ^3^, ^4^, ^5^ ^]^ far lower than the commercial level of about 10 mg cm^−2^ (hundreds of micron thick), resulting in areal capacitance from less than tens of millifarads per square centimeter to 200 millifarads per square centimeter at the best.^[^ ^2^, ^4^ ^]^ There is a pressing need to increase the thickness of the electrodes to be able to improve the areal capacitance. But it has been challenging to fabricate such high‐mass‐loading microelectrodes in a limited footprint.

Thick three dimensional (3D) electrodes with high aspect ratio could be a promising strategy to overcome this drawback, however, to date, few 3D microelectrodes were fabricated properly.^[^ ^6^, ^7^, ^10^, ^16^, ^17^, ^18^, ^19^, ^20^, ^21^, ^22^, ^23^, ^24^, ^25^ ^]^ Additive manufacturing technologies such as screen printing, ink jet printing, extrusion printing are promising for fabricating microelectrodes,^[^ ^17^, ^18^, ^19^, ^20^, ^21^, ^22^, ^26^ ^]^ however, the configurations of the electrodes are restricted by the spread nature of the ink or slurry. The fabricated electrodes usually end up with small thicknesses and big intervals, which reduces the areal energy density of the microsupercapacitors. Electrophoretic deposition of carbon nanoparticles or electrochemical polymerization of conductive polymers onto the pre‐patterned current collectors were also used to fabricate microelectrodes^,[^ ^5^, ^23^ ^]^ yet the processes were time‐consuming and the deposition or growth rates decrease along with the electrode thickness, prohibiting a proper increase of mass loading. Laser induced conversion of certain materials such as graphene oxide (GO)^[^ ^12^, ^24^, ^25^ ^]^ and polyimide (PI)^[^ ^15^, ^22^, ^27^ ^]^ were employed to straightforwardly generate graphene microelectrodes, which were much high‐throughput compared with the others,^[^ ^28^ ^]^ but the mass loading was still low due to the photothermal and/or photochemical effects could not take place at further depth without damage of the already generated graphene layer. Laser cutting of NiCo_2_S_4_‐nickel foam allowed for finger heights of up to 1200 µm with millimeter scale of finger width and interspace,^[^ ^10^ ^]^ however, the volume ratio of the NiCo_2_S_4_ layer to the porous nickel foam was much small (far less than 1%), trading off the advantage of compactness for MSCs. Embedding active carbon or multiwall carbon nanotubes (MWCNTs) into the preformed interdigital microchannel of submillimeter depth were ever employed for high mass loading of microelectrodes,^[^ ^6^, ^7^, ^16^, ^29^ ^]^ however, the walls of microchannel blocked the ion diffusion pathway, resulting in moderate areal capacitance even at rather low scanning rates, such as 134 mF cm^−2^ at 5 mV s^−1^ using the 250 µm thick active carbon microelectrodes,^[^ ^7^ ^]^ or 19.5 mF cm^−2^ at 2 mV s^−1^ using the 190 µm thick MWCNTs microelectrodes.^[^ ^6^ ^]^

To manufacture high energy/kinetic MSCs with thick 3D electrodes, the electrical and mechanical stability of the electrodes need to be improved. Due to the absence of separator between microelectrode pairs, in‐plane microelectrodes usually had a messy issue of short circuit, caused by the deformation, exfoliation or lift‐off of microelectrode fingers during fabrication, package or usage. With the increase of mass loading, as the height and aspect ratio of the microelectrodes increased, the situation could get worse. The mechanical properties could be improved to a great extent if more binders were used for preparing electrode materials. However, extra binders do not only decrease the electrical conductivity significantly but also reduce the utilization of electrode surface area by separating or islanding the nanoparticles. Developing a new strategy that could improve the mechanical property of the electrodes, meanwhile maintaining or even improving the electrochemical performance of the MSC is critical.^[^ ^30^ ^]^

Based on our understanding on the dependence of mechanical and electrical properties on the correlation of the gel electrolyte and porous electrodes, we developed new 3D thick electrodes for MSCs by first filling gel electrolyte into porous electrode films, then defining the electrodes by laser ablation. Infilling gel electrolyte optimized the mechanical and electrical properties of the pre‐stacked porous electrode films, which enabled the laser ablation of 3D MSCs with not only high mass loading and high aspect ratio, but also high surface area utilization, fast ion diffusion, high robustness. The filled gel electrolyte in this way acted as binder, ion diffusion channel, and ion reservoir at the same time. The optimized 3D MSCs show great electrochemical and mechanical performance. The areal capacitance of our 3D microelectrodes was able to increase linearly with mass loading (or thickness) up to at least 13 mg cm^−2^ (or 260 µm) for a value of up to 4640 mF cm^−2^ based on active carbon. Our 3D MSCs delivered areal energy density up to 1318 μWh cm^−2^, comparable to the best of Li‐ion 3D microbatteries. The capability of MSCs delivering battery‐level areal energy density will likely meet the demands of electrochemical energy storage for IoTs and electronics. More importantly, further improvements in energy density may be achieved if a better active material is being used, for example, the high specific capacitance pseudocapacitance active materials (MnO_2_,^[^ ^31^ ^]^ V_2_O_5_,^[^ ^32^ ^]^ et al.).

## 2 Results and Discussion

### 2.1 Tuning Mechanical and Electrical Properties

**Figure 1:**
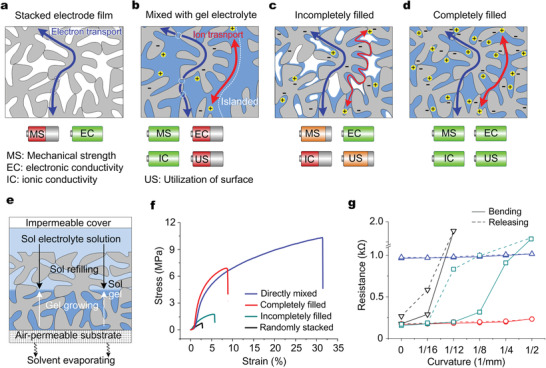
Tuning mechanical and electrical properties. The dependence of mechanical and electrical properties on the presence of gel electrolyte in pores. A comprehensive comparison of improved mechanical strength (MS), high electronic conductivity (EC), high ionic conductivity (IC) and efficient utilization of surface (US) between different electrode films: a) the thickly stacked electrode nanoparticles had poor mechanical strength (MS); b) the mixed gel electrolyte improved the MS but degenerated the electron conductivity (EC) and utilization of surface (US); c) the incompletely infilling of gel electrolyte degenerated the ionic conductivity (IC), MS and US; d) the completely infilling of gel electrolyte succeeded in those aspects. e) Bottom‐up infilling of gel electrolyte into the internal pores of electrode film. Curves for tensile strain versus stress f) and curvatures versus resistance g) at bending (solid curves) and releasing (dashed curves) process for MWCNTs films with different presence of gel electrolyte.

**Figure** **1** shows the dependence of mechanical and electrical properties on the correlation of gel electrolyte and porous electrode materials. Infiltration of gel electrolytes into internal pores of pre‐stacked electrode films could be helpful for both mechanical properties and electrical conductivity by bonding the electrode nanoparticles while unchanging their electrical contact (Figure 1d), compared with the ones with directly stacked electrode nanoparticles (Figure 1a) or the ones with directly mixed binders (Figure 1b). However, when the thickness of microelectrodes increased above certain values, for example above 3 µm to 5 µm for the carbon nanotubes films,^[^ ^6^, ^13^, ^33^ ^]^ the gel electrolyte such as PVA/H_3_PO_4_ could not well fill the under pores, especially for ones of micro or mesoscales. The incompletely filling of gel electrolyte resulted in voids in pores, which not only weakened the bonding effect, but also partially prevented the formation of electrode/gel electrolyte interfaces, declining the utilization of electrode surface for charge storage, and additionally tortured the ion diffusion pathway to reduce the ionic conductivity (Figure 1c).

For a combination of improved mechanical properties, high electronic and ionic conductivity, and efficient utilization of electrode surface area, it is necessary to completely fill the internal pores of thickly stacked electrode films with gel electrolyte (Figure 1d). Using our previously demonstrated bottom‐up infiltration method,^[^ ^8^ ^]^ as illustrated in Figure 1e, gel electrolytes were filled into thick electrode films (Figure S1, Supporting Information). Generally, the evaporation of solvent in electrolyte solution was forced downward through the bottom air‐permeable substrate (curved arrows), during which the gelation started from the bottom of electrode film instead of the top of sol solution and the gel grew upwards (white arrows) with continuously solvent evaporating and sol refilling (black straight arrows) until a complete.

Here, we used gel electrolyte PVA/H_3_PO_4_ and MWCNTs as model materials to provide evidence for the dependence of mechanical and electrical properties on different presence of gel electrolyte in pores of electrode films. After a completely infilling of gel electrolyte PVA/H_3_PO_4_ using the bottom‐up process, the fracture strength of a 300 µm thick MWCNTs film was tested as 6.9 MPa at the strain rate of 8.6% (Figure 1f, red), more than 8 times higher than the value of 0.8 MPa at the strain rate of 2.7% before infilling gel electrolytes (Figure 1f, black). The electrical conductivity was measured as 4.31 ×10^3^ S m^−1^, nearly same with the value of 4.32×10^3^ S m^−1^ before infilling gel electrolyte, significantly higher than the value of 7.6×10^2^ S m^−1^ for the MWCNTs film with mixed 20 wt% gel electrolyte. More importantly, after infilling gel electrolyte, the MWCNTs films did not show any crack even at a bending radius of as small as 1 mm (Figure S2c, Supporting Information), where the electrical resistance was nearly unchanged after releasing the bend (Figure 1f, red). As comparisons, before infilling gel electrolyte, the randomly stacked MWCNTs films showed many cracks at a bending radius of 16 mm and broken into debris at a bending radius of 1 mm (Figure S2a, Supporting Information). The MWCNTs films with mixed gel electrolyte showed cracks free but high resistance (Figure 1g, blue). These results indicated that the trade‐off relationship between mechanical strength and electric conductivity of the model electrode films was broken up after infilling gel electrolytes into their internal pores.

It is worth noting that the amount and concentration of electrolyte solution also influenced the filling of gel electrolyte. A better filling status could be achieved when more and diluted electrolyte is used, at a cost of longer time and thicker residue gel layer (as detailed in the supplementary information, Figures S3 and S4, Supporting Information). Here, the amount and concentration were optimized as about 0.3 mL cm^−2^/100 µm and 1 g PVA: 0.8 g H_3_PO_4_: 15 mL ultrapure water.

### 2.2 Laser Architecting 3D MSCs

**Figure 2:**
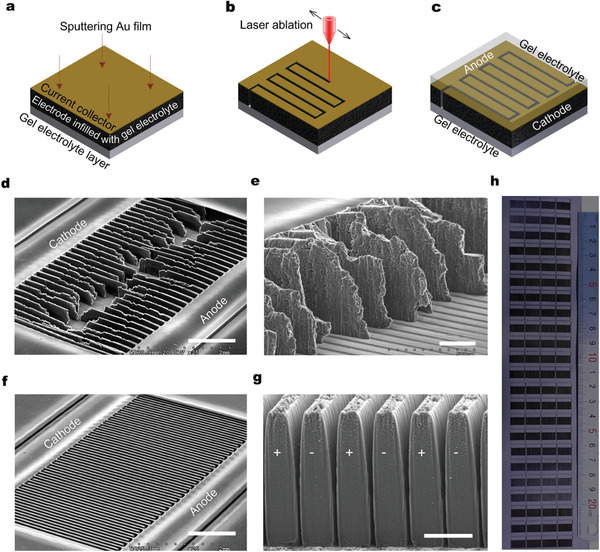
Laser architecting of 3D MSCs. Schematics for formations of current collector by sputtering an Au layer onto the surface of electrode film infilled with gel electrolyte (a), 3D microelectrode by laser ablation (b), and 3D microsupercapacitors after filling of gel electrolyte in and on the 3D microelectrodes (c). SEM images of microelectrode from incompletely infilled electrode film at title view (d) and cross view (e). SEM images of in‐plane 3D microelectrodes generated from completely infilled electrode films at tilted view (f) and cross views (g). Scale bars are 1 mm for (d,f), 250 µm for (e), and 100 µm for (g). (h) Snapshot of an electrode film with 92 pairs of 3D microelectrodes by laser ablation within 50 minutes. The area of each 3D microelectrode pairs was 5 mm × 10 mm. The thickness of 3D microelectrodes was about 200 µm.

**Figure** **2** shows the fabrication of 3D MSCs through laser ablation of the model electrode films with tuned electrical and mechanical properties. The geometrical sizes of microelectrodes features were defined by a laser marking machine with an optimized power of 3 W for avoiding short circuit while minimizing the width of ablated channels for less mass loading loss (Figure S5, Supporting Information). At a low power such as less than 2 W, it was difficult to cut through the thick electrode film (more than 500 µm) even with the repeated laser scanning, always resulting in microelectrodes with short circuit. With the increase of power, both the width and depth of the laser ablated channels increased. For instance, the opening width of ablated channels increased from about 20 µm at 1 W to 44 µm at 5 W, meaning an approximately doubled mass loss of 3D microelectrodes. Besides, at a high power such as above 5 W, the microelectrodes tend to be broken due to the increased thermal shock.

Through programming laser ablation of the 300 µm thick MWCNTs film with completely infilled gel electrolyte, in‐plane 3D microelectrodes were fabricated with a mass loading of up to 22 mg cm^−2^ and aspect ratios of up to 5 for microelectrode fingers, or 20 for interspaces, where the average width of fingers and interspaces were about 60 µm and 15 µm, respectively (Figures 2f, g). The mass loading and aspect ratios were higher than the previously reported by at least one order of magnitude (Table S1, Supporting Information).^[^ ^4^, ^5^, ^7^, ^10^, ^11^, ^12^, ^17^ ^]^ As a comparison, when the marking laser with the same power was scanning into the MWCNTs films with incompletely infilled gel electrolyte or the directly stacked MWCNTs films, the microelectrode features tended to crack, collapse, break or splash, disabling the generation of high aspect ratio 3D microelectrodes (Figures 2d,e and Figure S6b,c, Supporting Information). The formation of our high aspect ratio 3D microelectrodes benefited from the greatly improved fracture strength and strain rate, which allowed for the microelectrode features withstanding well with the thermal and mechanical disturbance from laser shock. In our experiments, 92 pairs of 3D microelectrodes were fabricated within 50 minutes (Figure 2h), suggesting a low cost and high efficiency of a micro‐fabrication process, compared with most of the others such as lithography, electroplating or electrodeposition, and various printing techniques.^[^ ^17^, ^18^, ^19^, ^20^, ^21^, ^22^, ^23^ ^]^ 3D MSCs were obtained by filling gel electrolytes into the interspace between cathode and anode, as shown in Figure S7, Supporting Information.

### 2.3 Electrochemical Analysis on the Presence of Gel Electrolyte

**Figure 3:**
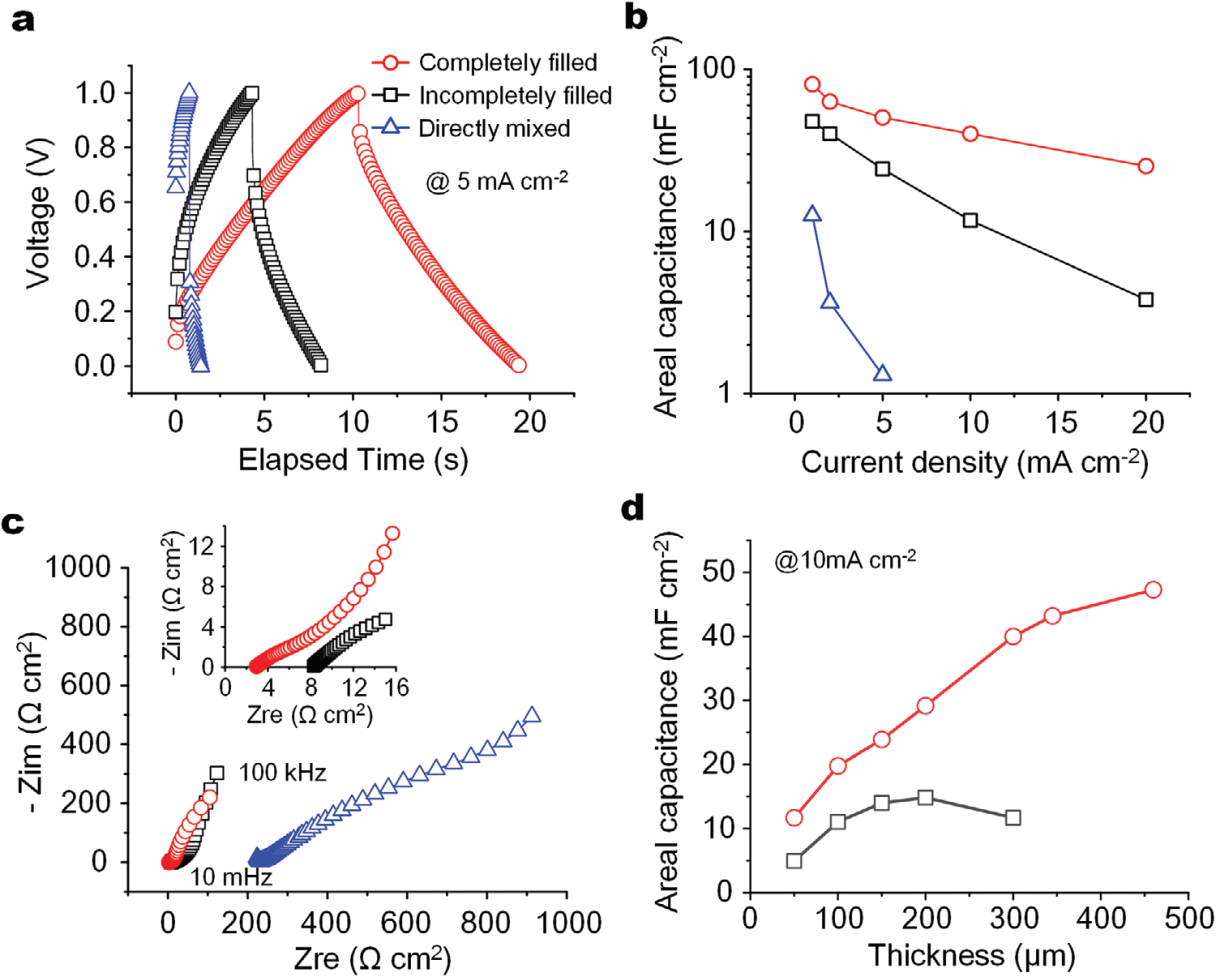
Electrochemical performances on different presence of gel electrolytes. a) GCD curves of 3D MSCs with different presence of PVA/H_3_PO_4_ in MWCNTs electrodes, including the completely infilled (red), the incompletely infilled (black), and the directly mixed (blue). b) Areal capacitance versus current densities for the 3D MSCs at current densities of 1 ‐ 20 mA cm^−2^ and voltage window of 0 ‐ 1 V. c) Nyquist plots of the 3D MSCs at frequencies from 100 kHz to 10 mHz. The inset in (c) indicates the corresponding curves at high frequency. d) Areal capacitance versus thickness of 3D MSCs at the same current density of 10 mA cm^−2^.

**Figure** **3** shows the different electrochemical performances of 3D MSCs, depending on the different presence of gel electrolyte in the internal pores of porous electrodes. The 3D MSCs were fabricated with the same geometrical sizes: microelectrode fingers’ width, height and interspaces were 400 µm, 300 µm and 15 µm, respectively. We cycled those 3D MSCs by galvanostatic charge‐discharge (GCD) at the same current density of 5 mA cm^−2^, and observed the longer elapsed time and a smaller IR drop for the one with completely infilled gel electrolyte (Figure 3a, red), compared with the incompletely infilled (Figure 3a, black) or the directly mixed (Figure 3a, blue), suggesting an increased areal capacitance and a decreased ESR. In addition to high areal capacitance, the improved capacitance retention was also observed (Figure 3b, Figure S8, Supporting Information), for example from 80.8 mF cm^−2^ at 1 mA cm^−2^ to 25.3 mF cm^−2^ at 20 mA cm^−2^ with retention of 31.3% (Figure 3b, red), compared with the corresponding smaller values of from 47.7 mF cm^−2^ to 3.8 mF cm^−2^ with retention of only 7.9% for the incompletely infilled one (Figure 3b, black). The 3D MSCs based on the mixed structures of MWCNTs and gel electrode exhibited the worst capacitance and capacitance retention, and almost could not be charged at current density higher than 5 mA cm^−2^ (Figure 3b, blue), which was mainly ascribed to the poor conductivity and inefficient utilization of MWCNTs surface. The different ESR of 3D MSCs with different filling status was confirmed by Nyquist plot (Figure 3c), showing a value of 2.9 Ω cm^2^ for the completely infilled structure, compared with 8.3 Ω cm^2^ for the incompletely infilled structure, or 223.6 Ω cm^2^ for the mixed structure, as obtained from the intersection between the real axis and the curves.

These improved electrochemical performances for 3D MSCs with completely infilled gel electrolyte structures originated from the void‐free electrode/gel electrolyte interfaces, which enabled an efficient utilization of internal pores surface for maximizing EDL sites, compared with the counterparts. The void‐free electrode/gel electrolyte interfaces further optimized the ion diffusion pathway without bypassing any voids, which enabled the high capacitance retention and the reduced ESR. Consequently, the areal capacitance was able to linearly increase with the thickness of microelectrodes up to at least 345 µm (Figure 3d, red, Figure S9a, Supporting Information) at a high current density of 10 mA cm^−2^, which was critical for improving areal energy density of MSCs via increasing mass loading. As a comparison, for the 3D MSCs with incompletely infilled gel electrolyte, the areal capacitance was not only inferior but also ceased increase for thickness above about 200 µm (Figure 3d, black, Figure S9b, Supporting Information). Before our work, it was usually difficult to linearly increase the areal capacitance with mass loading or thickness of microelectrodes. For example, the areal capacitance ceased the linear increase at a value of 18 mF cm^−2^ above thickness of about 2 µm for microelectrodes of carbide‐derived carbon (CDC) using electrolyte of tetraethylammonium tetrafluoroborate (TEABF_4_) in acetonitrile,^[^ ^3^ ^]^ at a value of 1.4 mF cm^−2^ above thickness of about 5 µm for microelectrode of SWCNTs using electrolyte of PVA/H_3_PO_4_,^[^ ^33^ ^]^ at a value of 50 mF cm^−2^ above four printed layers of rGO foam using electrolyte of PVA/H_2_SO_4_.^[^ ^17^ ^]^

### 2.4 Electrochemical Analysis on Geometrical Sizes

**Figure 4:**
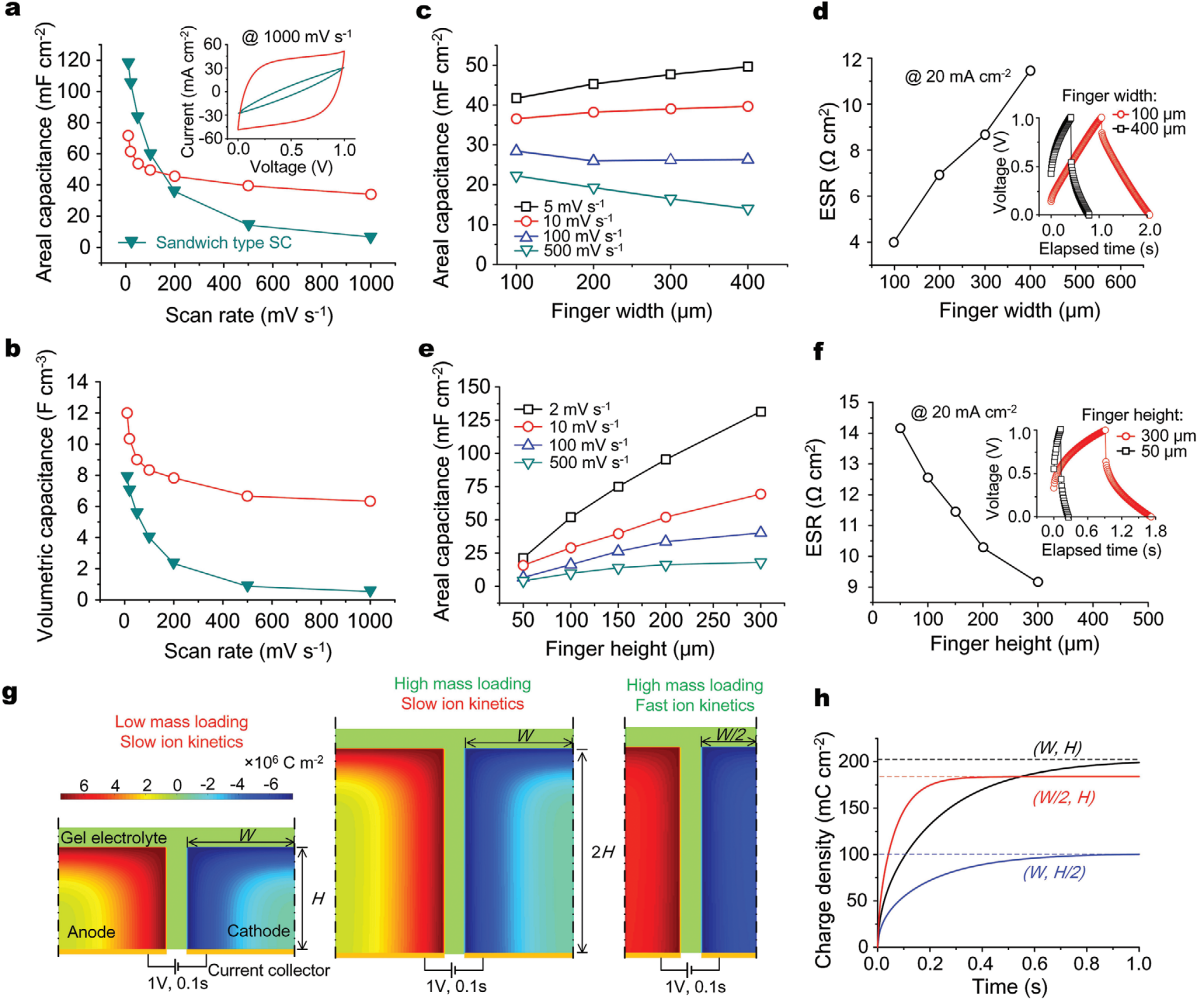
Electrochemical analysis on geometrical sizes of microelectrodes fingers. Areal capacitance (a) and volumetric capacitance (b) versus voltage scanning rates of 10–1000 mV s^−1^ for 3D MSC (red) and sandwich type supercapacitors (cyan). The inset in (a) shows the CV curves at 1,000 mV s^−1^. The areal capacitance versus finger widths (c) or heights (e) at the different voltage scanning rates. ESR versus finger widths (d) or heights (f), confirmed by the IR drops in GCD curves (insets) at the given current density of 20 mA cm^−2^. Note that the finger heights were constant as about 150 µm when varying widths (b,c), the finger widths were constant as about 400 µm when varying the finger heights (e,f), and the interspace between microelectrode fingers was constant as about 15 µm when varying widths or heights. (g) Snapshots of EDL distribution in half cycles of symmetric in‐plane microelectrode pairs with varying heights (*H or H* /2) and widths (*W*, or *W* /2), which were charged by a constant voltage of 1 V at 0.1 s. (h) Curves for areal ion density versus time absorbed in a microelectrode fingers with different width and heights, as denoted.

We further compared 3D MSCs with the sandwich type supercapacitors to investigate the electrochemical roles of 3D microelectrodes (**Figures** **4**a,b). We cycled a 3D MSC, whose microelectrodes height, width and interspace were 300 µm, 60 µm and 15 µm (Figure 2f,g), respectively, and observed nearly ideal rectangle shape of cyclic voltammetry (CV) curves at a high voltage scanning rate of 1,000 mV s^−1^ (**Figure** **4**a, inset), much superior to that for the counterpart using the same thick electrode films with well‐infilled gel electrolyte. The areal capacitance for the sandwich type supercapacitors seemed higher than that of 3D MSC at small voltage scanning rates such as lower than 100 mV s^−1^, due to the almost doubled mass loading of electrode materials. However, the volumetric capacitance and capacitance retention for electrode materials in sandwich type supercapacitors were obviously lower than that in 3D MSC at the wide range of voltage scanning rates, 10 ‐ 1,000 mV s^−1^ (Figure 4b, Figure S10, Supporting Information). For example, the capacitance retention of 3D MSC was 47%, higher than 6% of the sandwich type supercapacitors (Figure S11, Supporting Information). The high rate capability and high volume capacitance mainly benefited from the fine microelectrode fingers and small interspacing (Figure 2f), which shortened the ion diffusion pathway in and between microelectrode fingers for fast ionic kinetics.

Figure 4c–f shows electrochemical performances dependence of 3D MSCs on the geometrical sizes of 3D microelectrode fingers. At relatively high rates, the areal capacitance of 3D MSCs tended to decrease with finger width (Figure 4c, black, red), for instance from 23 mF cm^−2^ for 100 µm width down to 14 mF cm^−2^ for 400 µm width, which was confirmed by CV curves at 500 mV s^−1^ (Figure S12a, Supporting Information). However, at low voltage scanning rates such as below 10 mV s^−1^, the varying tendency of areal capacitance reversed: it increased with finger widths (Figure 4c, blue, cyan). This increase of areal capacitance was caused by different factors. Firstly, the mass loading of microelectrode increased with finger width because of the increased duty ratio (*k*) between finger width and interspace. Furthermore, the low voltage scanning rates, corresponding to a long time for ion diffusion, allowed for ions to sufficiently diffuse through the entire width of microelectrode fingers for the formation of more EDL sites. Figure 4d shows that the ESR of 3D MSCs increased from 3.97 Ω cm^2^ to 11.45 Ω cm^2^ with finger width increasing from 100 to 400 µm, which were calculated from the IR drops in GCD curves at 20 mA cm^−2^ (Figure 4d, inset). The increase of ESR was mainly ascribed to the increased ion diffusion pathway through a wide microelectrode finger compared with that through a narrow one (Figure 4h).

Figure 4e shown that the areal capacitance increased almost linearly with a continuous increase of microelectrode height up to at least 300 µm at a wide range of voltage scanning rates from 10 mV s^−1^ to 500 mV s^−1^, which can be intuitively observed from the CV and GCD curves of 3D MSCs in Figure S13, Supporting Information. It was notable that the ESR decreased, instead of increased, with finger heights (Figure 4f), different from that when increasing finger widths (Figure 4d). The decrease of ESR and increase of areal capacitance with microelectrode finger height might due to the equivalent parallel effects of microelectrodes finger with small height.

For better understanding the size functions of microelectrode fingers, we showed an insight into the EDL behaviors in microelectrodes by finite element method (FEM) (Figure 4g,h). Figure 4g showed snapshots of constant‐voltage charging, which indicated that the EDL sites formed from the surface to the center. At a certain moment before reaching the steady state of charging, more portion of electrode materials were charged in narrow and high microelectrode fingers (Figure 4g, right) than that in wide, high ones (Figure 4g, middle) and wide, low ones (Figure 4g, left). Consequently, the areal EDL density in narrow, high microelectrode fingers was higher than that in wide, low microelectrode fingers at the same time, as compared between the red and black curves, or between black and blue curves in Figure 4h, respectively. Generally, it needs a short time to sufficiently form EDL sites in narrow microelectrodes than that in wide ones, corresponding to the fast ion kinetics; the narrowed microelectrodes coupled with a big height, corresponding to high aspect ratio, benefited both ion kinetics and mass loading. These geometrical sizes‐dependent EDL behaviors provided general guidance for designing the feature sizes of 3D microelectrodes: (i) narrow, high fingers allowed for 3D MSCs to exhibit larger areal capacitance at higher charging rate with smaller ESR, which was important for an increase of both areal energy density and power density; (ii) wide, high fingers resulted in larger areal capacitance but an increased ESR, corresponding to higher areal energy density but a compressed power density.

### 2.5 Universality and Practical Consideration

**Figure 5:**
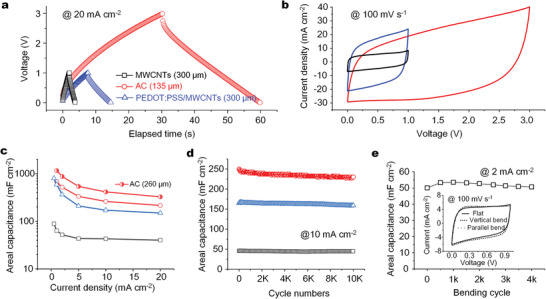
Universality for different materials. GCD curves at 20 mA cm^−2^ (a) and CV curves at 100 mV s^−1^ (b) of 3D MSCs based on different electrode and gel electrolyte materials. Areal capacitance versus current densities (c) or charging–discharging cycle numbers (d) of 3D MSCs using the different materials. (e) The areal capacitance versus bending cycles, confirmed by GCD curves at 2 mA cm^−2^ after every 500 cycles at bending radius of 2 mm. The inset in (e) shows the CV curves of a MWCNTs 3D MSC at different bending states, including vertical and parallel bend to the microelectrode fingers versus flat state.

To demonstrate the possible universality of our design, additional electrode and electrolyte materials, including about 300 µm thick pseudocapacitive composite electrode films of poly(3,4‐ethylenedioxythiophene) polystyrene sulfonate (PEDOT:PSS)/MWCNTs, up to 260 µm thick active carbon films, and ionic liquid‐based gel electrolyte of PVDF‐HFP/[BMIM]BF_4_ were used to fabricate 3D MSCs successfully. The typical GCD (**Figure** **5**a) and CV (Figure 5b) curves of 3D MSCs using active carbon and PVDF‐HFP/[BMIM]BF_4_, PEDOT:PSS/MWCNTs and PVA/H_3_PO_4_, or MWCNTs and PVA/H_3_PO_4_ exhibited symmetric triangle shapes and rectangle shapes, respectively, generally indicating the good performances.

The 3D MSCs based on PEDOT:PSS/MWCNTs delivered an areal capacitance of up to 800 mF cm^−2^ at 0.5 mA cm^−2^ for the entire device considering the area of microelectrode fingers and interspace (Figure 5c, Figure S14, Supporting Information). The high areal capacitance benefited from the pseudocapacitive behavior of PEDOT as well as the improved conductivity of the composite film, up to 1.23×10^4^ S m^−1^, much higher than 4.32×10^3^ S m^−1^ for the stacked MWCNTs film or 1.46×10^2^ S m^−1^ for the PEDOT:PSS film. The value of areal capacitance was at least 4 times higher than the state‐of‐the‐art^[^ ^7^, ^12^, ^13^, ^14^, ^15^, ^34^ ^]^ (Table S1, Supporting Information): 74.3 mF cm^−2^ at 5 mV s^−1^ for 3D MSCs with 1500 µm thick 3D microelectrodes of printed assembly graphene,^[^ ^17^ ^]^ 134 mF cm^−2^ at 5 mV s^−1^ for the ones with 250 µm thick 3D microelectrodes of microcavities‐defined active carbon,^[^ ^7^ ^]^ 180.5 mF cm^−2^ at 0.5 mA cm^−2^ for the ones with 101 µm thick 3D microelectrodes of laser‐induced graphene (LIG)‐MnO_2_.^[^ ^15^ ^]^

Using the most commonly used commercial electrode material active carbon, as well as ionic liquid‐based gel electrolyte of PVDF‐HFP/[EMIM]BF_4_ for a wide voltage window of 0 – 3 V, the 3D MSCs of different thicknesses were fabricated and performed (Figure 5a,c, Figure S15, Supporting Information). The areal capacitance almost linearly increased with thickness increasing from 80 µm to at least 260 µm at current density below 10 mA cm^−2^. With the increase of current density, the capacitance trended to cease increase with thickness and even decline at current density as high as 100 mA cm^−2^. Specifically, the areal capacitance for the 260 µm thick 3D MSCs was up to 1160 mF cm^−2^ (4640 mF cm^−2^ for electrode) at current density of 1 mA cm^−2^ and maintained capacitance retention of 36% and 18% at current density of 10 mA cm^−2^ and 50 mA cm^−2^ (Figure S16, Supporting Information), respectively.

These values of areal capacitance coupled with the wide voltage window were folds higher than the previously reported results (Table S1, Supporting Information), although various novel electrode materials were employed in their microelectrodes, such as sulfur‐doped graphene,^[^ ^11^ ^]^ LIG/MnO_2_,^[^ ^15^ ^]^ MnO_2_/Au,^[^ ^31^ ^]^ graphene/polyaniline (PANI),^[^ ^14^ ^]^ PANI nanofibers,^[^ ^35^ ^]^ NiCo_2_S_4_‐carbon nanofiber (CNF).^[^ ^10^ ^]^ The superiority of our 3D MSCs was mainly owing to the high‐mass‐loading and high‐aspect‐ratio 3D microelectrodes with micro‐scale structural adjustment of electrode materials and gel electrolyte, which could fast the ion kinetics and facilitate the efficient utilization of internal pores surface of electrodes.

The mechanical and electrochemical stability, self‐discharge and leakage current, as well as areal energy and power densities, are all important in practice. Our 3D MSCs were outstanding in those aspects. For example, the 3D MSCs showed excellent electrochemical stability with capacitance retention of 97%, 96.6% and 92.5% after 10000 charging‐discharging cycles at 10 mA cm^−2^ for the ones using electrode materials of MWCNTs, PEDOT:PSS/MWCNTs and active carbon, respectively (Figure 5d). The high cycling stability of the pseudocapacitive PEDOT:PSS/MWCNTs based 3D MSC, compared with the capacitance retention of 52% for the one based on PEDOT:PSS only (Figure S17, Supporting Information), might be owing to the high conductive and stable scaffold of MWCNTs in the composite of PEDOT:PSS/MWCNTs. We also tested the self‐discharging performances of the 3D MSCs based on PEDOT:PSS/MWCNTs (Figure S18, Supporting Information). After charging to a voltage of *V*_max_ = 1 V at 0.5 mA cm^−2^, the device exhibited an ultra‐small self‐discharging current from 184 pA cm^−2^ to 120 pA cm^−2^. The time of half‐voltage discharging, i.e., discharging from *V*_max_ to 1/2*V*_max_, was measured to be longer than 27 h, compared to that from 9 h to 21 h for commercial supercapacitors.^[^ ^12^ ^]^

The 3D MSCs were mechanically robust. We bent the MWCNTs‐based 3D MSCs vertically or parallelly to the microelectrodes fingers at a curvature radius of 2 mm, and observed CV curves with nearly the same rectangle shape as that at flat state (Figure 5e, inset), indicating outstanding flexibility. After 4000 times of bending and releasing, the capacitance retention was as high as 96%, suggesting outstanding mechanical durability (Figure 5e). This robustness of our 3D MSCs was owing to both the enhanced mechanical properties of electrode materials and the interesting construction, where the 3D microelectrodes were fully embedded in the gel electrolyte, providing mechanical protection to the internal microelectrodes (Figures S7b,d, Supporting Information). Thanks to the high flexibility, the 3D MSCs can be conveniently integrated with objects of uneven shapes. For example, three 3D MSCs in serials tandem could be conformally integrated with a cylindrical small glass bottle to power a blue LED (Figure S19a, Supporting Information).

The tandem MWCNTs‐based 3D MSCs performed the anticipated good GCD and CV curves (Figure S20, Supporting Information) and exhibited fast charging‐discharging capabilities. For instance, after being charged by a 3 V Li‐ion battery package within 2 seconds, the three 3D MSCs in serials could power 28 red LEDs in parallel for more than 10 seconds (Figure S19b, Supporting Information), or power a single blue LED for more than 160 seconds (Figure S19a, Supporting Information), where the footprint area of each microelectrode pattern in the 3D MSCs was only 0.5 cm^−2^. It is also notable that the tandem 3D MSCs devices could be conveniently self‐connected without additional connecting wires through programming the laser scanning pathway (Figure S19, Supporting Information).

**Figure 6:**
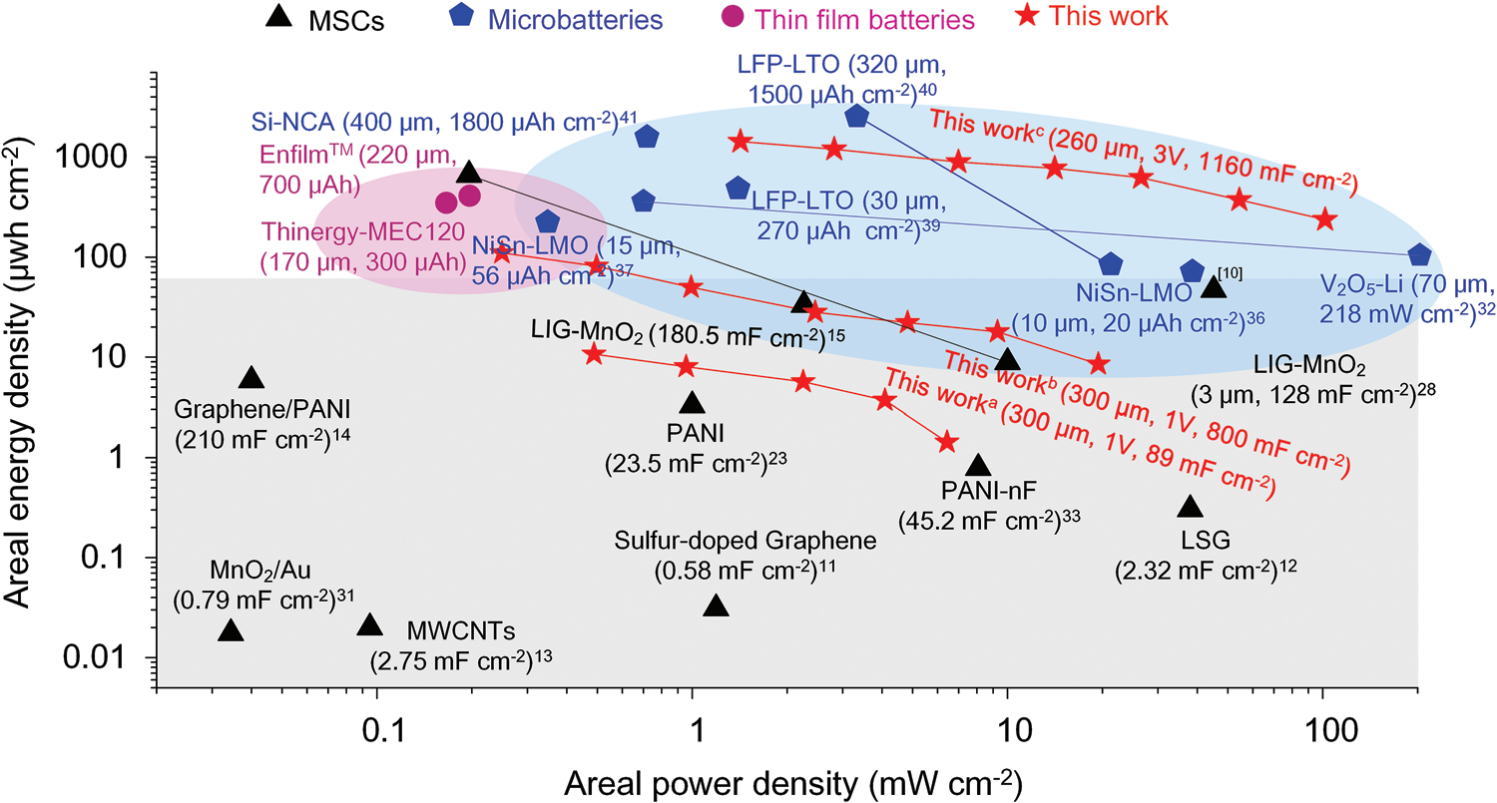
Areal‐normalized Ragone plot. The gray, violet, and blue regions refer to MSCs, Li‐ion thin‐film batteries, and Li‐ion 3D microbatteries, respectively. Note that this work^a^, this work^b^ and this work^c^ refer to our 3D MSCs based on MWCNTs, PEDOT:PSS/MWCNTs, and AC, respectively. Abbreviations: NiSn‐LMO: nickel–tin (anode) and lithium manganese oxide (cathode), LTO‐LFP: Li_4_Ti_5_O_12_ (anode) and LiFePO_4_ (cathode), LTO‐LCO: Li_4_Ti_5_O_12_ (anode) and LiCoO_2_ (cathode), LSG: laser scribe graphene, LIG: laser induced graphene.

Since the energy of a supercapacitor is proportional to the square of discharged voltage window, the 3D MSCs based on AC and ionic liquid‐based gel electrolyte delivered high areal energy densities at a wide voltage window of 0 − 3 V. The areal energy density reached up to 1318 μWh cm^−2^ (50.7 mWh cm^−3^) at 1.43 mW cm^−2^ (0.06 W cm^−3^), or 63.9 μWh cm^−2^ (2.46 mWh cm^−3^) at 102.99 mW cm^−2^ (3.96 W cm^−3^) with thickness of 260 µm, where the volumetric energy and power densities were detailed in Figure S21, Supporting Information. This areal energy was at least one order of magnitude higher than the state‐of‐the‐art^[^ ^11^, ^12^, ^13^, ^14^, ^15^, ^23^, ^31^, ^35^ ^]^ (**Figure** **6**, Table S1, Supporting Information). For example, the areal energy density was 0.3 μwh cm^−2^ at 38 mW cm^−2^ for MSCs based on microelectrode of laser scribed graphene (LSG),^[^ ^12^ ^]^ 12 μwh cm^−2^ at 1.3 mW cm^−2^ for the ones based on the microchannel defined 3D microelectrodes of active carbon,^[^ ^7^ ^]^ 32.4 μwh cm^−2^ at 2.33 mW cm^−2^ for the one based on 3D microelectrodes of LSG‐MnO_2_,^[^ ^15^ ^]^ and 49 μwh cm^−2^ at 45 mW cm^−2^ for the ones based on 3D microelectrodes of NiCo_2_S_4_‐CNF.^[^ ^10^ ^]^

This level of areal energy density has surpassed the commercially available Li‐ion thin‐film batteries, and is comparable to the best Li‐ion 3D microbatteries.^[^ ^36^, ^37^, ^38^, ^39^, ^40^, ^41^ ^]^ The commercial Li‐ion thin‐film batteries, Enfilm (220 µm, 700 μAh) and Thinergy‐MEC120 (170 µm, 300 μAh) for instance, delivered areal energy densities of 413.3 μWh cm^−2^ at 0.21 mW cm^−2^ and 362.7 μWh cm^−2^ at 0.18 mW cm^−2^, respectively. The areal energy density for the state‐of‐the‐art Li‐ion 3D microelectrodes was 65 μWh cm^−2^ at 36 mW cm^−2^ using the 10 µm thick holographic patterned 3D microelectrodes of NiSn‐LMO,^[^ ^36^ ^]^ 225 μWh cm^−2^ at 0.345 mW cm^−2^ using the 15 µm thick nanoporous microelectrodes of NiSn‐LMO,^[^ ^37^ ^]^ 270 μWh cm^−2^ at 0.32 mW cm^−2^ using the 30 µm thick microwall‐defined microelectrodes of LFP‐LCO,^[^ ^39^ ^]^ 2694 μWh cm^−2^ at 2.7 mW cm^−2^ using 320 µm thick 3D‐printed interdigital microelectrodes of LFP‐LTO,^[^ ^40^ ^]^ 1600 μWh cm^−2^ at 0.64 mW cm^−2^ using 400 µm thick sandwich type 3D microelectrodes of etched Si‐NCA.^[^ ^41^ ^]^ The latest 3D micro‐battery using 70 µm thick V_2_O_5_‐Li by nanoimprinting lithography showed areal energy density of 345 μWh cm^−2^ and peak power density of 75.5 mW cm^−2^ for the package cell and 218 mW cm^−2^ for the unpackage.^[^ ^32^ ^]^ Besides, most of those thin‐film batteries or 3D microbatteries usually suffered from drawbacks of low cycle life, complex package of the liquid electrolytes, or low power density, inferior to our 3D MSCs.

## 3 Conclusions

We demonstrated a design of 3D MSCs by tuning the mechanical and electrical properties of the commonly breakable porous electrode films, which allowed for laser architecting of 3D microelectrodes with high mass loading and high aspect ratio. The critical step was to combine the improved mechanical properties, high electrical conductivity, efficient utilization of surface area, and fast ionic kinetics through completely infilling gel electrolyte into porous electrodes. Using this method, the common electrodes and gel electrolytes had an ability to construct 3D MSCs for delivering uncommon areal energy density, comparable to the best of 3D Li‐ion microbatteries, which is very promising for promoting the development of IoTs.^[^ ^42^ ^]^ We believe our method for architecting 3D MSCs is broadly useful for different electrode and gel electrolyte materials because of the universality of infilling gel electrolyte into thick electrode films^[^ ^8^ ^]^ and laser ablation of the reinforced electrode films. We note that perhaps areal energy density even higher than the present best value (1318 μWh cm^−2^) may be possible using advanced electrode films, such as metal organic framework (MOF), Mxene, RuO_2_, graphene,^[^ ^43^, ^44^, ^45^, ^46^, ^47^ ^]^ if they can be stacked into thick films.

## 4 Experimental Section

### Infilling Gel Electrolyte into Electrode Films

1. *Preparing electrolyte solutions*: The PVA/H_3_PO_4_ electrolyte solution was prepared as follows. 1 g of PVA (Mw 95000 g mol^−1^, 95% hydrolyzed, Aldrich) was dissolved in 15 mL of ultrapure water at 90 °C with vigorous stirring until the solution became transparent. After cooling to room temperature, 0.8 g of H_3_PO_4_ (85% wt. aqueous solution, Aldrich) was added into the solution and stirred for 12 h at room temperature to form a homogeneous solution. The PVDF‐HFP/[EMIM]BF_4_ electrolyte solution was prepared as follows. 1g of PVDF‐HFP (average Mw ≈455000, Sigma‐Aldrich) was dissolved in 10 mL of acetone by stirring until the solution became transparent. Then 2 g of ionic liquid of [EMIM]BF_4_ (99% wt., Aladdin) was added into the solution and stirred for 12 h at room temperature to form a homogeneous solution.
2. *Infilling PVA/H_3_PO_4_ into MWCNTs films*: Randomly stacked free‐standing MWCNTs films were prepared by vacuum filtration of MWCNTs aqueous solution (9‐10% wt., Aladdin, diameter 40‐60 nm, length < 10 µm), where the thickness of MWCNTs films depended on the amount of the solution, followed by baking of the MWCNTs film at 150 °C for 2 h to reduce the electrical resistance. Next, the MWCNTs film was placed onto a PVDF millipore membrane (0.22 µm pore size, Fisher Scientific), which was pre‐located on a loose sponge. A slight overdose of sol electrolyte, typically 0.3 mL cm^−2^/100 µm, was cast onto the MWCNTs film. Next, a 60 µm thick PET film was placed on top of the electrolyte solution. After 24 h at room temperature to downwards evaporate the water solvent, a MWCNTs film with completely infilled of gel electrolyte was obtained by tearing off the PVDF substrate and the PET cover. A MWCNTs film with incompletely infilled gel electrolyte was obtained by drop casting electrolyte solution on to MWCNTs films without a cover on the top to allow for solvent evaporation upwards directly. The MWCNTs films were further dried at 60 °C for 12 h.
3. *Infilling PVA/H_3_PO_4_ into PEDOT:PSS/MWCNTs films*: 18 mL PEDOT/PSS aqueous solution (1.3% wt., Aldrich) was mixed with 1 mL of MWCNTs aqueous solution (9‐10% wt., Aldrich, diameter 40‐60 nm, length < 10 µm) by vigorous stirring under 60 °C for 3 h to form a composite solution. The free‐standing PEDOT:PSS/MWCNTs films were prepared by drying the composite solution in a petri dish at normal temperature and pressure, where the thickness of films depended on the amount of the solution, followed by baking at 120 °C for 20 mins to form PEDOT:PSS/MWCNTs electrode films. The PEDOT:PSS/MWCNTs electrode films were completely infilled with gel electrolyte using the same process as described above in section (2).
4. *Infilling PVDF‐HFP/[EMIM]BF_4_ into active carbon films*: 0.5 g PVDF (HSV900, Arkema) was firstly dissolved into 120 mL N‐Methyl pyrrolidone (NMP, > 99 %, Aladdin) to form a homogeneous solution by stirring for 3 h in a glovebox. 8 g active carbon (AC, YEC‐8B) and 1.5 g carbon black (BP2000, CABOT) were mixed up by magnetic stirring and then dried under 120 °C for 10 h. The carbon mixture was added into the PVDF solution in an agate jar, followed by milling for 24 h, obtaining a well‐dispersed active carbon slurry. Microhole arrays were fabricated in the aluminum foil (22 µm in thickness, AFT ELECTRONIC CO., LTD, Guangdong, China) by laser ablation (Figure S22, Supporting Information), working as evaporation channels and current collector. The carbon slurry was then coated on the microhole‐arrayed aluminum foil via doctor blading, followed by baking at 60 °C for 12 h in a glovebox to evaporate the solvent of NMP, forming active carbon electrode film with microhole‐arrayed current collector. Typically, 0.4 mL cm^−2^/100 µm solution of PVDF‐HFP/[EMIM]BF_4_/acetone was cast onto the active carbon film and an impermeable 50 µm thick Kapton film was placed on the top of the electrolyte solution. After 24 h at room temperature to evaporate the solvent downwards through the microhole array in the aluminum foil, an active carbon film with completely infilled gel electrolyte was obtained by tearing off the cover of Kapton film.

### Laser Ablation of 3D MSCs

For improving conductivity, the PEDOT:PSS/MWCNTs and MWCNTs electrode film with completely or incompletely infilled gel electrolyte, as well as the mixed film of 80 wt% MWCNTs and 20 wt% PAV/H_3_PO_4_ were sputtered with 100 nm thick Au layer (Discover635, DENTON VACUUM) as current collector before subject to a focused scanning laser, a Nd: YAG laser marking platform (3 W, wavelength of 1064 nm, Hans’ Laser). The microhole‐arrayed aluminum foil worked as current collector for the electrode of active carbon films. To avoid the water absorption of ionic liquid [EMIM]BF_4_, the laser ablation of infilled active carbon film was carried out by placing the infilled active carbon film in a sealed and transparent glass container. The laser scan path for an interdigital pattern was controlled by a computer. For a complete removal of the electrode materials in the laser scanning pathway, a repeat laser scanning was used. After the 3D microelectrodes were fabricated, drops of electrolyte solutions were cast onto the microelectrodes, followed by evaporation of solvent at room temperature for 24 h and then further drying at 60 °C for 12 h.

### Tests of Mechanical and Electrical Properties

MWCNTs electrode films with different presence of gel electrolyte, including the ones with completely and incompletely infilled PVA/H_3_PO_4_ gel electrolyte, the one with mixed gel electrolyte of PVA/H_3_PO_4_ and the one with randomly stacked MWCNTs film without gel electrolyte, were cut into samples with the same size of 20 mm × 5 mm. The thicknesses of those films were the same as about 300 µm. Both ends of each sample were separately fixed to each chuck of a tensile test machine (TY8000, JiangSu TianYuan Co., Ltd). The data of tensile strain versus stress was recorded by a computer when the samples were stretched at a speed of 2 mm min^−1^. The conductivity of the samples was tested by four probe method. The electric resistance of the samples at different curvature was recorded by a source‐meter (Keysight B2900A), where the curvatures were operated by a linear motor.

All electrochemical tests, including CV, GCD, and EIS, were carried out in a two‐electrode system using an electrochemical working station (Versastat 3, Princeton Applied Research, USA) at room temperature. The SEM images and EDS data were obtained by GeminiSEM 500 and Hitachi SU8010.

### Calculations

The areal capacitance for device (*C_A‐device_*) and electrode (*C_A‐electrode_*) was calculated according to the CV or GCD curves:

(2) $$C_{A-device}=\frac{\int i\mathrm{dv}}{2AV_{window}\cdot v}$$

(3) $$C_{A-device}=\frac{I\cdot \Delta t}{AV}$$

(4) $$C_{A-electrode}=4\cdot \frac{k+1}{k}\cdot C_{A-device}$$

where *V_window_*, *v* and *A* refer to the potential window, voltage scan rate, and geometric surface area of the devices including both electrode fingers and gaps, respectively;*I*,Δ*t*, and *V* refer to the current density, discharge time, and potential window excluding IR drop. *k* refers to the duty ratio for microelectrode finger width to the interspace.

The areal energy density (*E_A_*), power density (*P_A_*) and *ESR* for entire device were calculated as:

(5) $$E_{A}=\frac{C_{A-device}\cdot V^{2}}{2\cdot 3600}$$

(6) $$P_{A}=\frac{3600E_{A}}{\Delta t}$$

(7) $$ESR=\frac{\Delta V_{IR}}{2I}A$$

where Δ*V_IR_* is the potential value for IR drop.

The volumetric energy density (*E_v_*) and power density (*P_v_*) were calculated as:

(8) $$E_{v}=E_{A}\times \frac{10000}{H}$$

(9) $$P_{v}=P_{A}\times \frac{10000}{H}$$

where *H* is the total thickness of micro electrode in micron, including electrode materials and current collector.

### Statistical Analysis

All the electrochemical results were the average level of over five independent measurements. Capacitances were normalized to per square centimeter considering the typical geometric size of MSC for 0.5 cm^−2^(5 mm × 10 mm).

## Conflict of Interest

The authors declare no conflict of interest.

## Author contributions

C.L., X.L., and Q.Y. contributed equally to this work. J.S. and X.L. conceptualized the idea and designed the experiments. J.S. supervised the project. C.L. and X.L. prepared the electrode films, gel electrolytes, fabricated 3D MSC devices, and tested the electrochemical performances. C.L. and J.S. tested and analyzed the mechanical performances of samples. X.L. and Q.Y. analyzed the EDL behaviors in 3D microelectrodes. X.L., C.L., Q.Y., P.S., Y.W., and L.W. analyzed the electrochemical performances of 3D MSCs. All authors commented on experimental results. X.L., C.L., and J.S. co‐wrote the manuscript.

## Supporting information

Supporting Information

## Data Availability

The data that supports the findings of this study are available from the corresponding authors upon request.
