# Supplementary material for: Tuning the Mechanical and Electrical Properties of Porous Electrodes for Architecting 3D Microsupercapacitors with Batteries‐Level Energy
Source: Adv Sci (Weinh). 2021 Jun 20;8(15):2004957. doi: 10.1002/advs.202004957 (PMC8336509; doi:10.1002/advs.202004957)
Supplement: Supplementary file 1 — Supporting Information [file ADVS-8-2004957-s001.pdf]

WILEY-VCH

© 2021 Wiley-VCH GmbH

ADVANCED SCIENCE
Open Access

Supporting Information

for *Adv. Sci.,* DOI: 10.1002/advs.202004957

Tuning the mechanical and electrical properties of porous electrodes for architecting 3D microsupercapacitors with batteries-level energy

Congming Li, Xiangming Li, Qingzhen Yang, Pengcheng Sun, Lifeng Wu, Bangbang Nie, Hongmiao Tian, Yingche Wang, Chunhui Wang, Xiaoliang Chen,* and *Jinyou Shao**

Supplementary Materials for

Tuning the mechanical and electrical properties of porous electrodes for architecting 3D microsupercapacitors with batteries-level energy

Congming Li, Xiangming Li, Qingzhen Yang, Pengcheng Sun, Lifeng Wu, Bangbang Nie, Hongmiao Tian, Yingche Wang, Chunhui Wang, Xiaoliang Chen, Jinyou Shao**

Correspondence to: xiangmingli@xjtu.edu.cn; jyshao@xjtu.edu.cn

This PDF file includes:

Figures S1 to S22

Table S1

Supplemental reference list

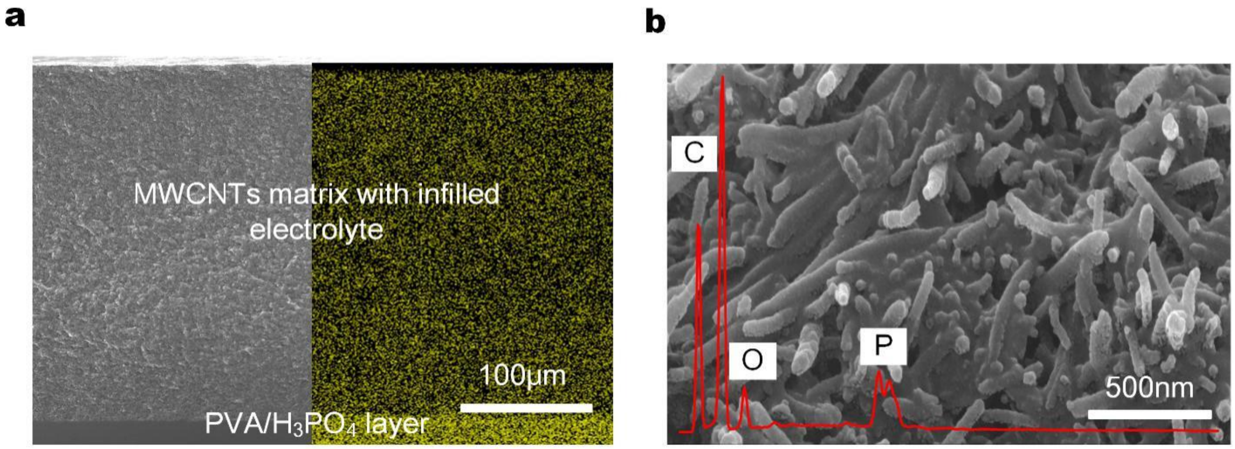

Figure S1 | MWCNTs electrode film with completely infilled PVA/H_3PO_4. (**a**) SEM image for section view (left) and energy dispersive spectroscopy (EDS) mapping scanning of phosphorus (right) of the MWCNTs film with infilled PVA/H_3PO_4. The uniform distribution of phosphorous (yellow dots) indicating a well infilling of PVA/H_3PO_4. (**b**) High resolution SEM image of the MWCNTs film with infilled PVA/H_3PO_4. Overlaid is the EDS curve where the phosphorous peaks indicate the presence of PVA/H_3PO_4.

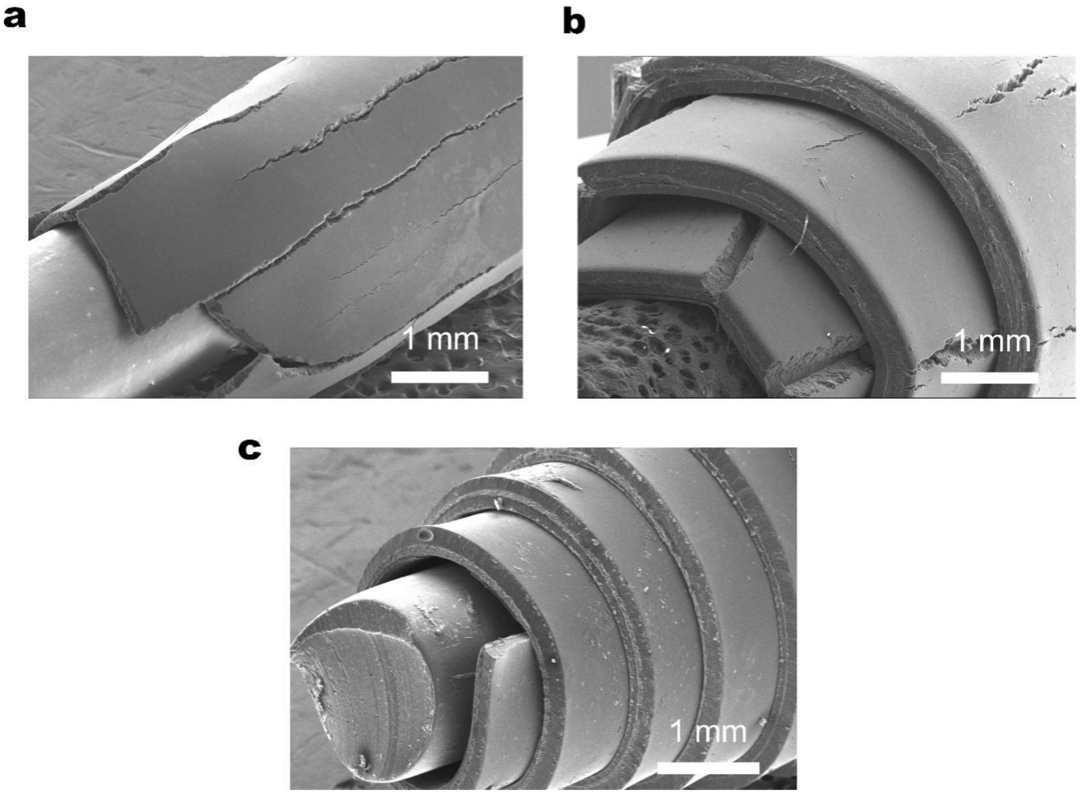

Figure S2 | The rolled different MWCNTs electrodes films with different presence of gel electrolyte in pores. SEM images of the stacked MWCNTs films without gel electrolyte (**a**), with incompletely infilled gel electrolyte of PVA/H_3PO_4 by the commonly (**b**), and with completely infilled gel electrolyte by bottom-up process (**c**), where the MWCNTs films were rolled over PVA wires with the same diameter of 2 mm.

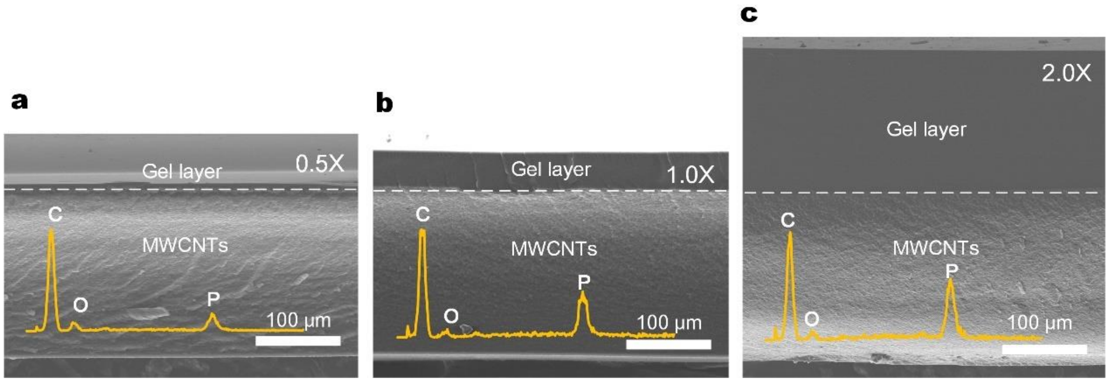

Figure S3 | The amount electrolyte influences the bottom-up filling. SEM images of MWCNTs film infilled with different amount PVA/H_3PO_4/H_2O electrolyte: (a) 0.5×, (b) 1.0× and (c) 2.0×amount of electrolyte solution of 0.3 mL cm^{-2}/100μm. Overlaid on the images are EDS scans where the phosphorus peaks indicate the presence of the electrolyte. It was notable that with the increase of electrolyte amount, the intensity of phosphorus increased, indicating that more electrolyte solution benefited the filling of gel electrolyte. However, the residual gel layer on electrode film got significantly thick for the doubled amount with only a slight increase of phosphorus peaks compared with the one with normal amount; while less amount such as 0.15 mL cm^{-2}/100 μm could not supply sufficient gel electrolyte for filling, resulting in an incompletely filling. Those results suggest a completely filling of gel electrolyte required an amount of at least. Here, the amount was optimized as about 0.3 mL cm^{-2}/100 μm.

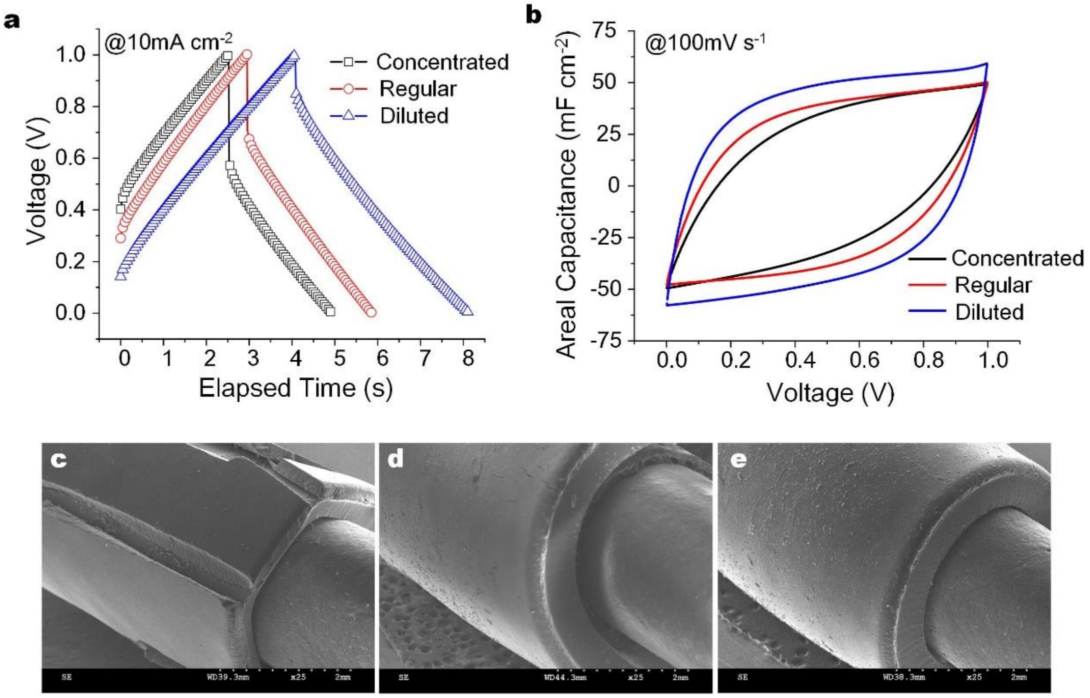

Figure S4 | The electrolyte concentration influences the bottom-up filling. GCD curves at 10 mA cm^{-2} (a) and CV curves at 100 mV s^{-1} (b) of MSCs based on MWCNTs films infilled with different electrolyte concentration. SEM images of the rolled-up MWCNTs films, which were infilled with doubled (c), normal (d) and halved (e) concentrations of electrolyte solution. Note the tube radius was 1 mm and thickness of MWCNTs films were about 220 μm.

In our experiment, for keeping a constant of final gel electrolyte material, the amount of solution was used as 0.15 mL cm^{-2}/100μm, 0.3 mL cm^{-2}/100μm, and 0.6 mL cm^{-2}/100μm for the doubled, normal and halved concentration, respectively. The diluted electrolyte allowed for better infiltration as confirmed by better electrochemical (a, b, bule curves) and mechanical (c) performances compared with counterparts, but suffered from a drawback of a long filling time. The filling time was about 16 hours, 48 hours, and more than five days when the concentration was declined from the doubled one to the halved one. Finally, considering the filling status and efficiency, the concentration of electrolyte solution was optimized as 1 g PVA: 0.8 g H_3PO_4: 15 mL ultrapure water.

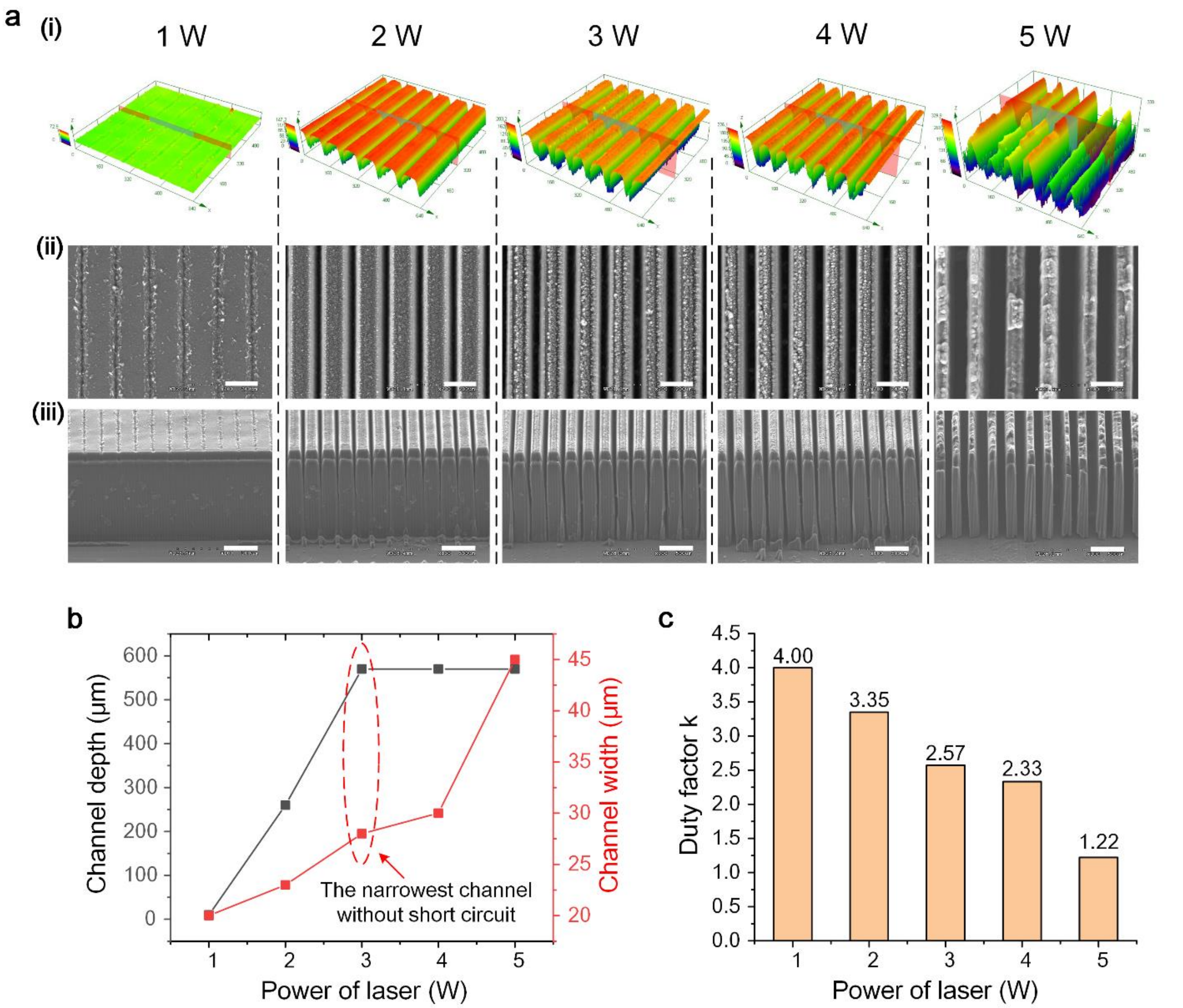

Figure S5 | Dependence for microelectrodes on power of laser. (a) 3D surface of microelectrodes characterized by laser scanning confocal microscope (line i), SEM images from top view (line ii) and cross view (line iii). (b) Statistics for depth and opening width of the laser ablated channels Vs laser power, and the corresponding duty factor *k* (the ration between microelectrode finger width and the channel width) (c). Note that the scale bar of (line ii) is 100 μm and 200 μm for (line iii), the thickness of electrode is about 570 μm.

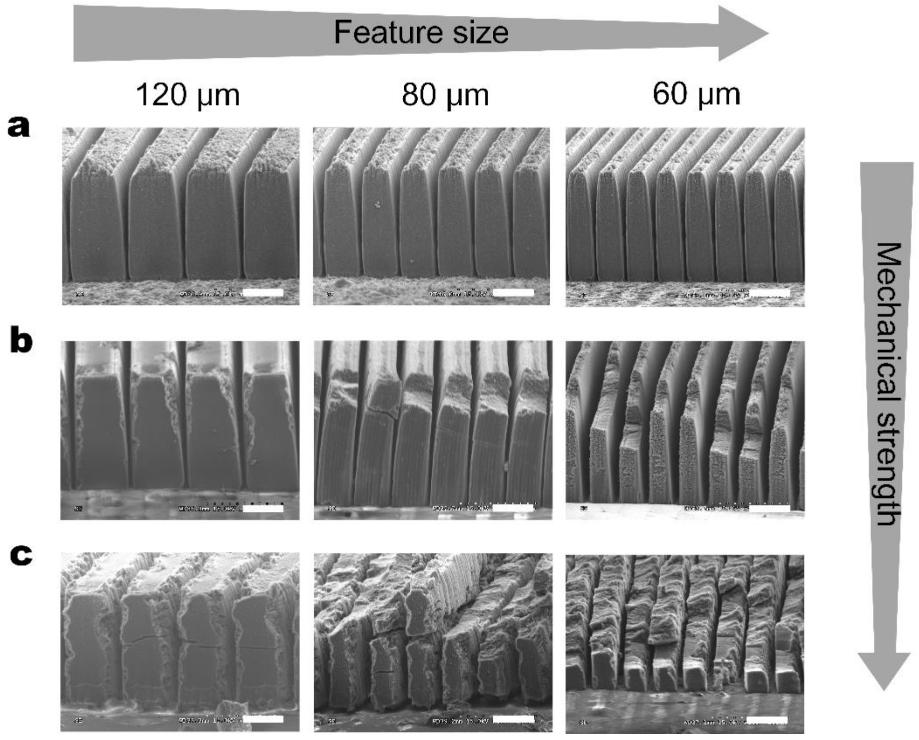

Figure S6 | Microelectrode features by laser ablation of MWCNTs electrode films with different filling status of gel electrolytes. From top to bottom rows: microelectrodes generated from the MWCNTs films with completely (row **a**) and incompletely (row **b**) infilled with gel electrolytes of PVA/H_3PO_4, and directly stacked MWCNTs films without gel electrolytes (row **c**). From left to right columns: the microelectrode finger width decreased from 120 µm (left column), 80µm (middle column) to 60 µm (right column) as denoted. Note that all of the microelectrodes have the same height of about 300 µm. Scale bars: 100 µm.

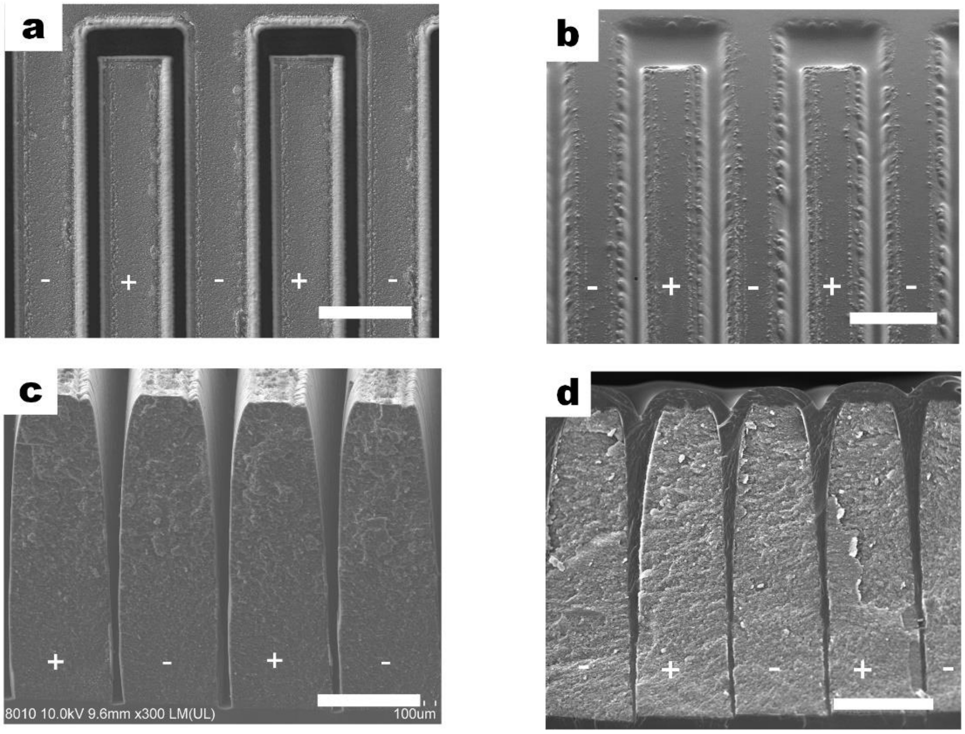

Figure S7 | Formation of 3D MSCs based on laser architecting**.** SEM images from cross view (**a, c**), top view (**b, d**) of 3D microelectrodes before and after filling gel electrolyte, respectively. Note the scale bars were 100 µm.

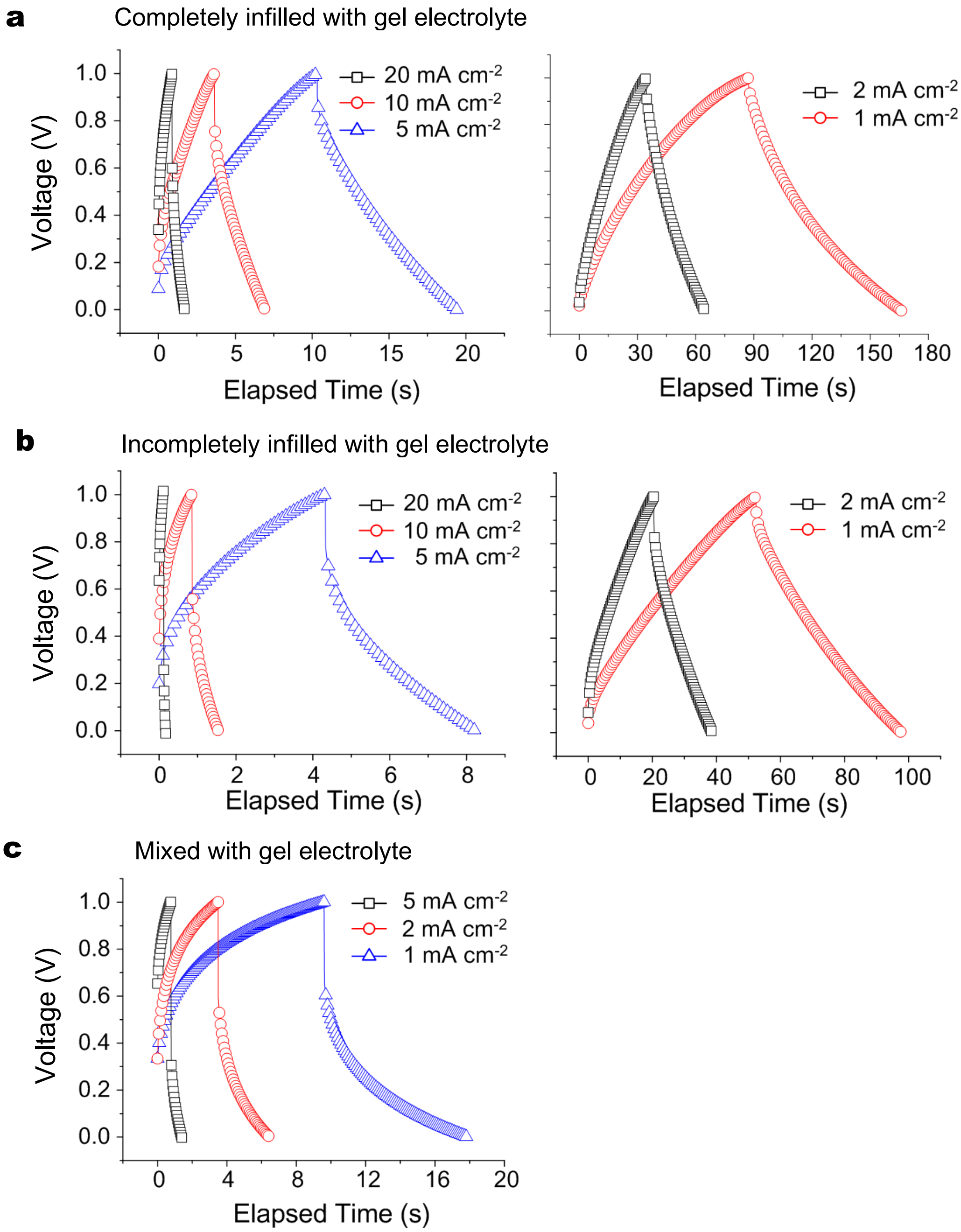

Figure S8 | GCD curves of 3D MSCs with different presence of gel electrolyte. GCD curves for MSCs with completely infilled gel electrolyte at current densities from 1 mA cm^{-2} to 20 mA cm^{-2} (**a**), incompletely infilled gel electrolyte at current densities from 1 mA cm^{-2} to 20 mA cm^{-2} (**b**), and mixed with gel electrolyte at current densities from 1 mA cm^{-2} to 5 mA cm^{-2} (**c**). Note the voltage window was 0 - 1 V, the electrode material was MWCNTs, and gel electrolyte was PVA/H_3PO_4.

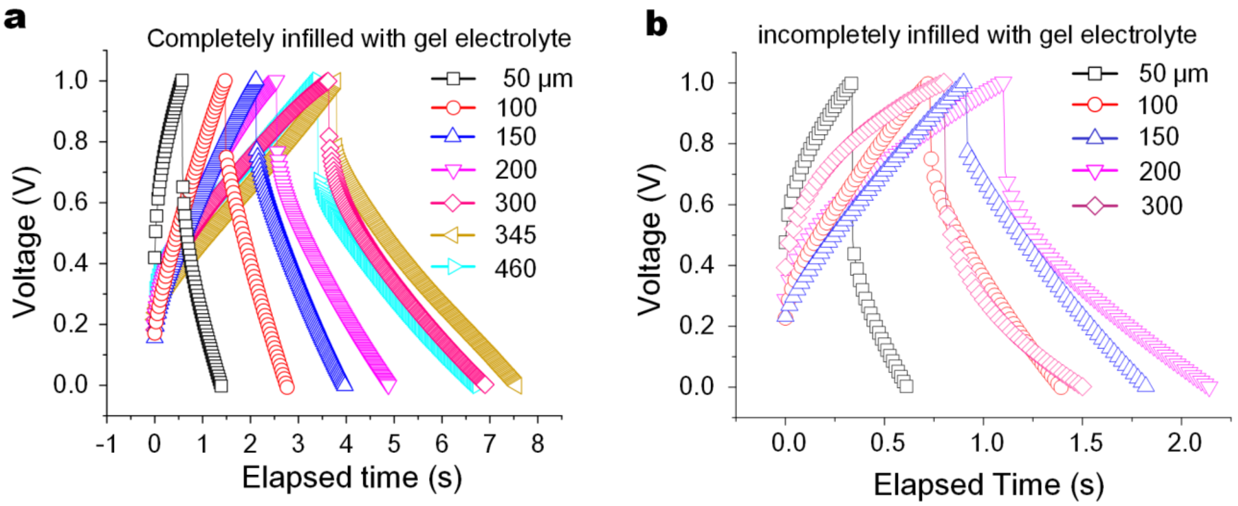

Figure S9 | GCD curves of 3D MSCs with different microelectrode thicknesses. GCD curves of 3D MSCs with completely infilled gel electrolyte for microelectrodes thickness from 50 μm to 460μm (**a**) or incompletely infilled gel electrolyte for microelectrodes thickness from 50 μm to 300 μm (**b**) at the same current density of 10 mA cm^{-2}.

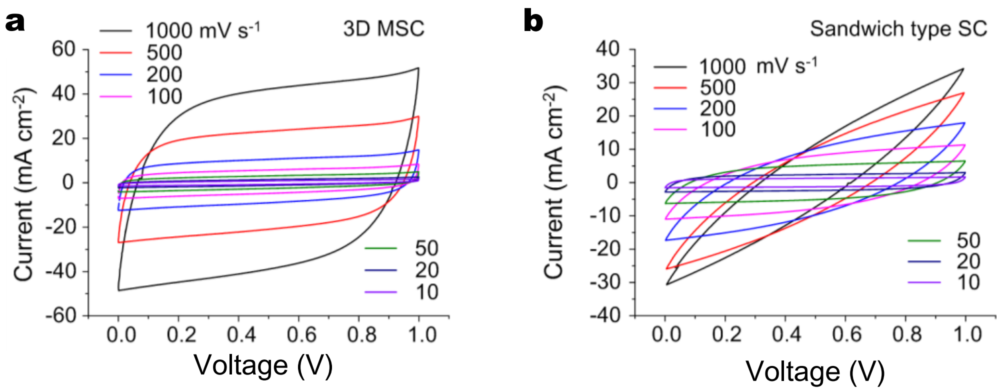

Figure S10 | CV curves of different types of supercapacitors. CV curves of a 3D MSC (**a**) and a sandwich type supercapacitor (**b**) at a wide range of voltage scanning rates from 10 mV s^{-1} to 1000 mV s^{-1}. Note that the MWCNTs films in both were completely infilled with gel electrolyte of PVA/H_3PO_4 and had the same thickness of 300 μm.

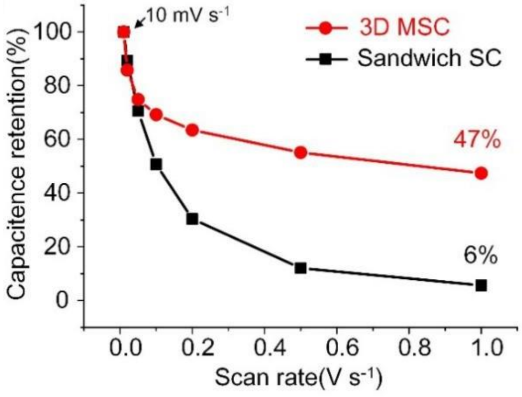

Figure S11 | Rate capabilities of 3D MSCs and sandwich supercapacitors. The voltage scanning rate increased from 10 mV s^{-1} to 1,000 mV s^{-1}. Note that the electrode films for MSCs and sandwich type supercapacitors are the MWCNTs films completely infilled with gel electrolyte of PVA/H_3PO_4 and had the same thickness of 300 μm.

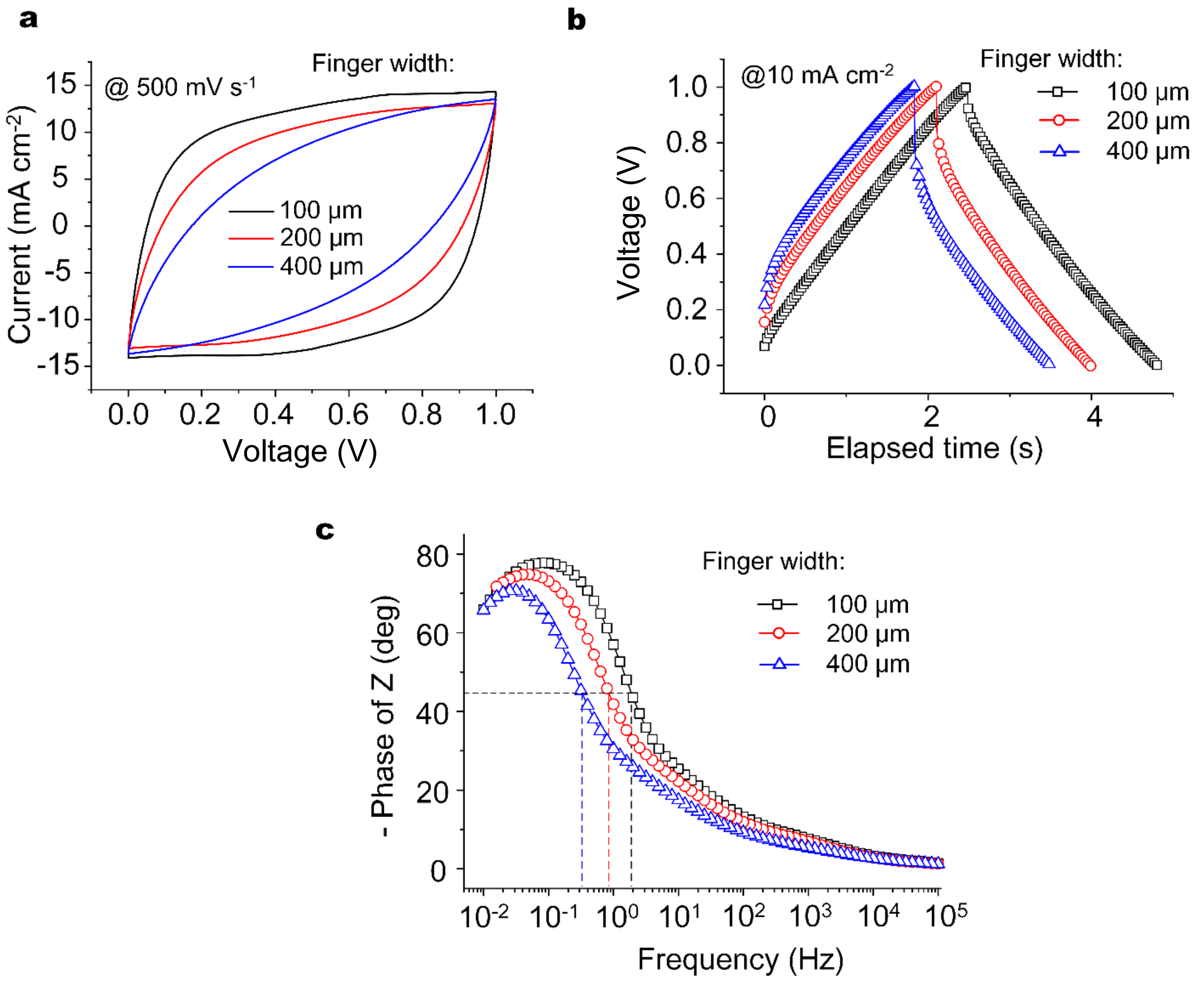

Figure S12 | The electrical performances of 3D MSCs vs microelectrode finger widths. CV **(a)** and GCD **(b)** curves of 3D MSCs with different finger widths at voltage scanning rates of 500 mv s^{-1} and current density of 10 mA cm^{-2}, respectively. **(c)** Bode plot of 3D MSCs with different finger widths, where the characteristic frequency varying with finger widths, 3D MSCs with the narrowest finger have the highest characteristic frequency, indicating fast kinetic behavior enabled by short diffusion pathway. Note the height of 3D microelectrodes were the same as 150 µm when varying the finger widths.

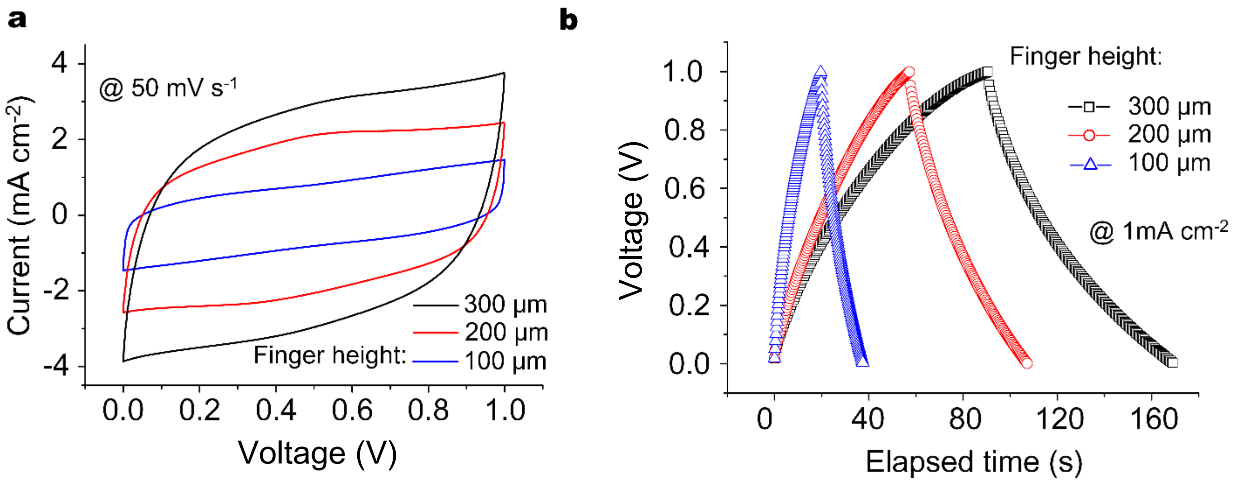

Figure S13 | The electrical performances of 3D MSCs vs microelectrode finger heights. CV **(a)** and GCD **(b)** curves of 3D MSCs with different finger heights at voltage scanning rates of 50 mv s^{-1} and current density of 1 mA cm^{-2}, respectively. The areal capacitance generally increased with finger height. Note the width of 3D microelectrodes were the same as 400 µm when varying the finger heights.

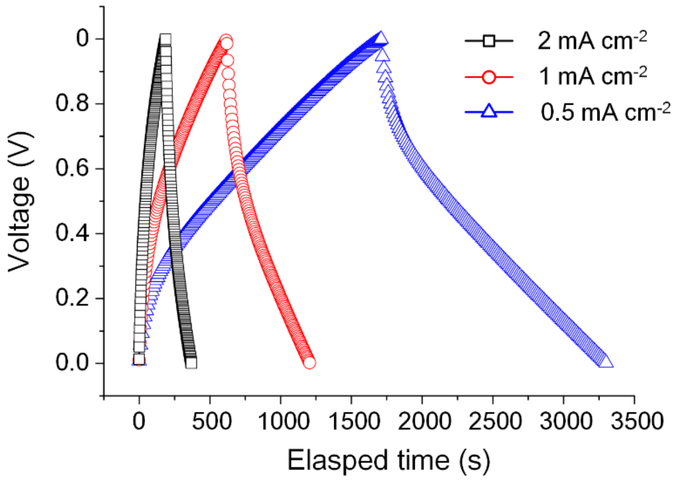

Figure S14 | GCD curves of a 3D MSC based on PEDOT:PSS/MWCNTs and PVA/H_3PO_4. Note the 3D microelectrode height width and interspace were 300 μm, 180 μm and 20 μm, respectively.

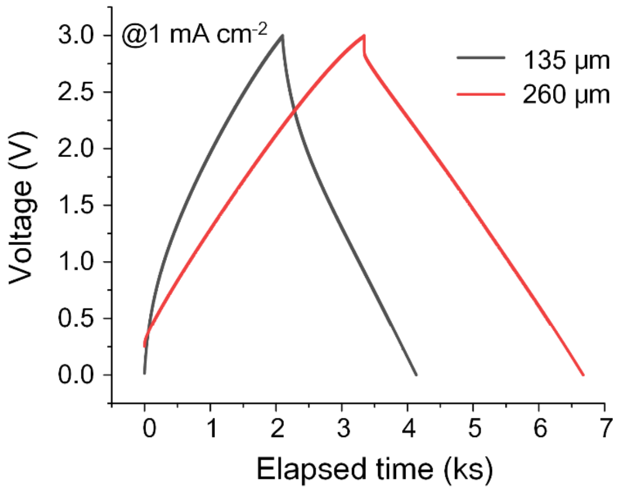

Figure S15 | GCD curves of 3D MSCs based on active carbon and gel electrolyte of PVDF-HFP/[EMIM]BF_4. Note the 3D microelectrode width and interspace were 180 μm and 20 μm, respectively, and the current density was 1 mA cm^{-2}.

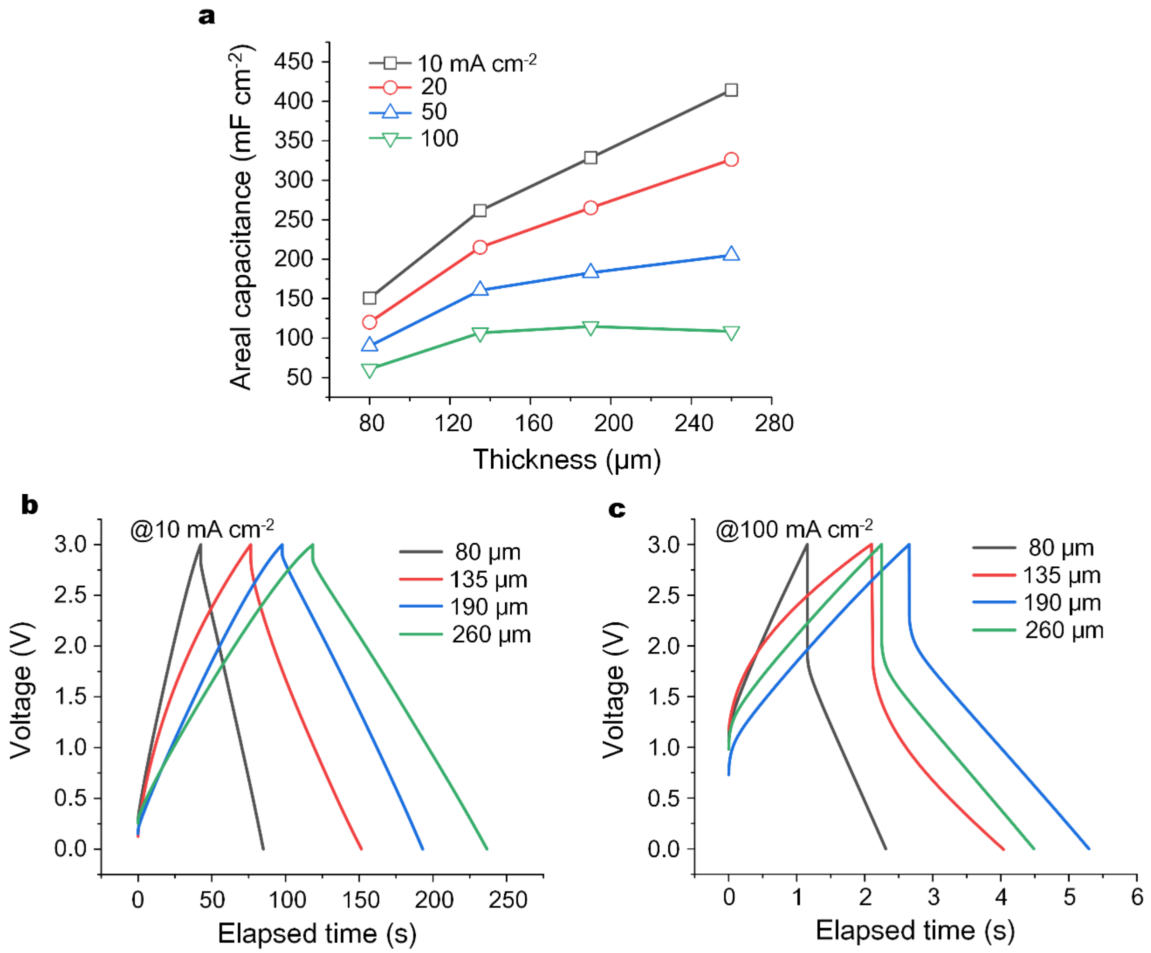

Figure S16 | Thickness dependence of MSCs based on AC and PVDF-HFP/EMIMBF$_4$. (a) Areal capacitance vs different thicknesses at different current densities. GCD curves at typical current densities of 10 mA cm^{-2} (b) and 100 mA cm^{-2} (c).

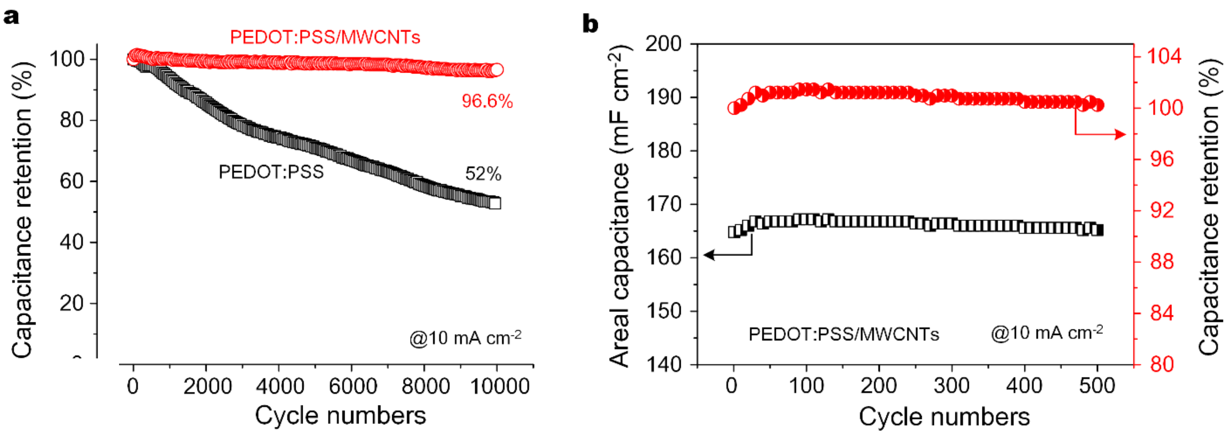

Figure S17 | Electrochemical cycling stability for 3D MSCs with different materials. The capacitance retention was as high as 96.6% for 3D MSCs based on PEDOT:PSS/MWCNTs, compared with 52% for the one based on PEDOT:PSS only, after 10,000 charging-discharging cycles at the same current density of 10 mA cm^{-2}.

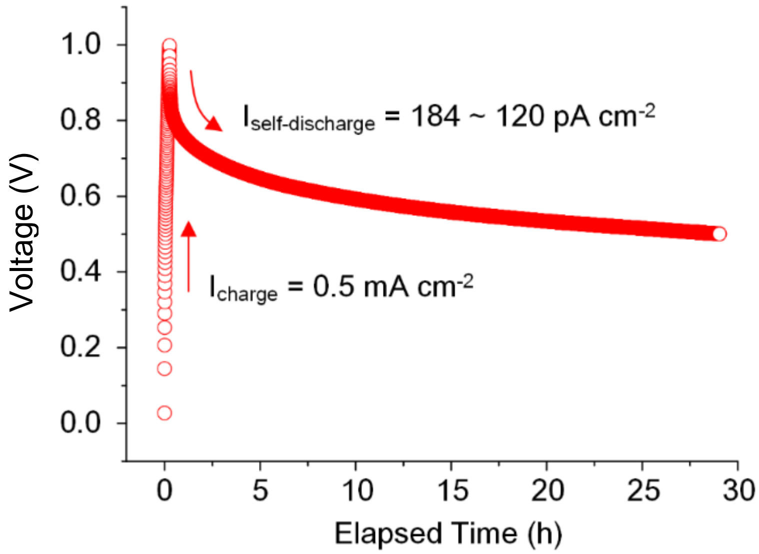

Figure S18 | Self-discharge of 3D MSCs based on PEDOT:PSS/MWCNTs. The device was charged up to a maximum voltage of 1 V at a current of 0.5 mA cm^{-2}. The time for self-discharge from V_{max} = 1 V to $1/2V_{max}$ =0.5 V was more than 27 h with a self-discharge current of 184 ~ 120 pA cm^{-2}.

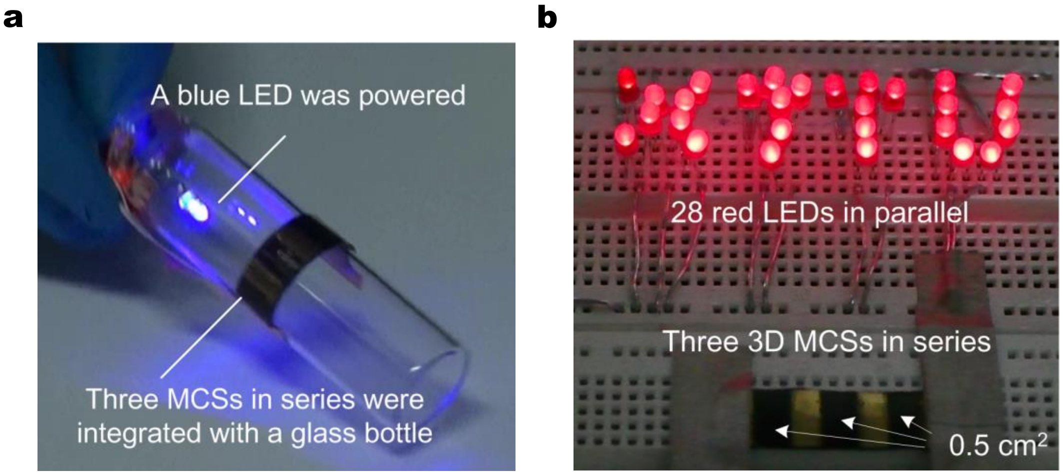

Figure S19 | Snapshots of 3D MSCs powering LED. Three serial tandem 3D MSCs were integrated with a glass bottle with a diameter of 2 cm, powering a blue LED after charged by a 3 V lithium battery within 2 seconds **(a)**. Three 3D MSCs in series powering 28 red LEDs in parallel **(b)**. Note the footprint area of each 3D microelectrodes was 0.5 cm^2.

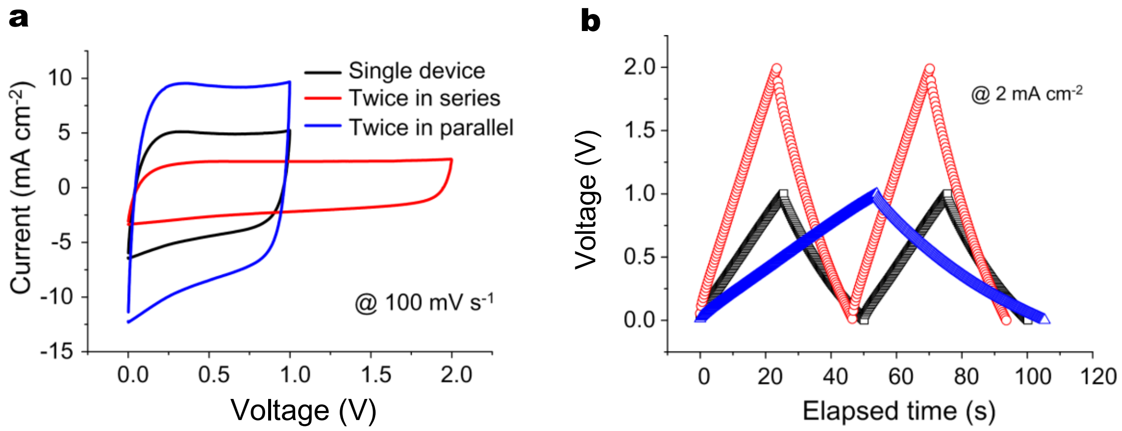

Figure S20 | CV and GCD curves of tandem 3D MSCs based on MWCNTs. The twice in series performed doubled voltage window of 0 - 2V and half of current (red, in **a** and **b**) compared with the single one (black, in **a** and **b**); the twice in parallel performed a doubled current and the same voltage window of 0 - 1V (blue, in **a** and **b**) compared with the single one.

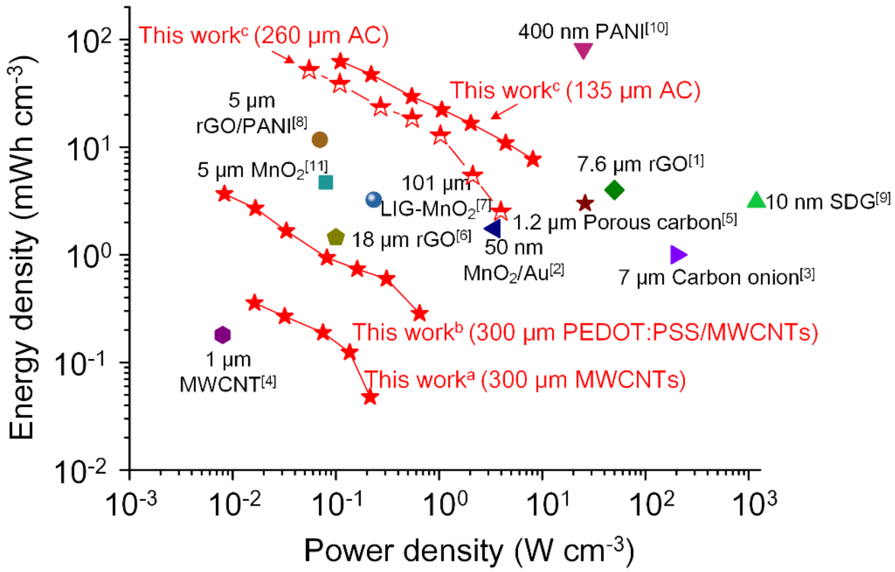

Figure S21 | Volumetric energy and power density comparations between MSCs[1-11]. Note that this work[a], this work[b] and this work[c] refer to our 3D MSCs based on MWCNTs, PEDOT:PSS/MWCNTs, and AC, respectively. Abbreviations: SDC: Sulfur-Doped Graphene; LIG: Laser-Induced Graphene.

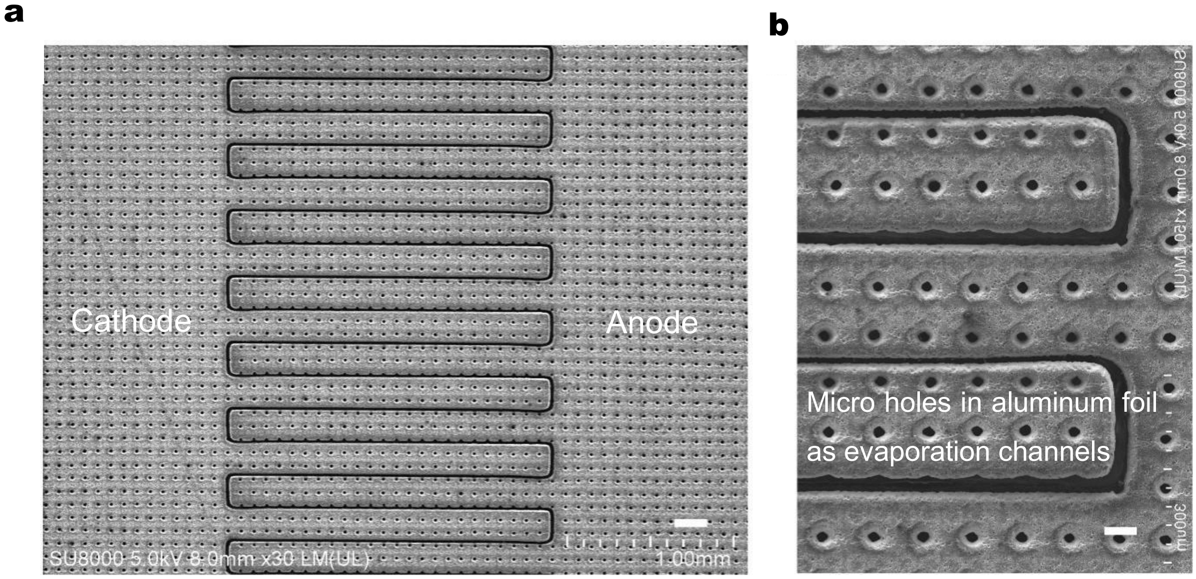

Figure S22 | Current collector for 3D microelectrodes based on active carbon. SEM images for top view (**a**) and high resolution (**b**) of microhole-arrayed aluminum current collectors. Note the micro hole array in the aluminum foil was fabricated by laser ablation before coating active carbon slurry, working as evaporation channels during the infilling of solid electrolyte. Scale bars: 200 μm (**a**), 50 μm (**b**).

Table S1 | Comparison between MSCs, 3D microbatteries and thin film batteries.

	Electrode materials	Thickness (μm)	Aspect radio	Electrolyte	Voltage (V)	Capacity (mF cm^{-2})	Power density (mW cm^{-2})	Energy density (uWh cm^{-2})	Stability	Ref.
**MSCs**	MWCNTs	300	5	PVA/H_3PO_4	0-1	74 @10 mV s^{-1}	6.43	17.89	10000/97%	**This work**[a]
	PEDOT:PSS/ MWCNTs	300	2	PVA/H_3PO_4	0-1	800 @ 0.5 mA cm^{-2}	19.42	110.2	10000/96.6%	**This work**[b]
	AC	135	0.75	PVDF-HFP /[EMIM]BF_4	0-3	681.9 @ 1 mA cm^{-2}	1.49 (109.3)	842.17 (103.84)	10000/92.5%	**This work**[c]
	AC	260	1.44	PVDF-HFP /[EMIM]BF_4	0-3	1160 @ 1 mA cm^{-2}	1.43 (102.99)	1318 (63.9)		**This work**[c]
	$NiCoS_4$-CNF	1200	NA	PVA/KOH	0-0.8	1000 @ 7mA cm^{-2}	45	49	10000/89%	[12]
	LIG-MnO_2	101	<0.1	PVA/LiCl	0-1	180.5 @ 0.5 mA cm^{-2}	2.334	32.4	6000/90%	[7]
	AC	250	0.83	PVA/H_3PO_4	0-1	134 @ 5 mV s^{-1}	1.3	12	10000/80%	[13]
	PANI-G	4.8	0.02	PVA/H_3PO_4	0-1	210 @10 mV s^{-1}	0.04	5.85	1000/92.6%	[14]
	mCel-membrane	NA	NA	[BMIM]Cl	0-1	153.34 @ 10 mV s^{-1}	4.45	1.24	1000/93%	[15]
	PANI nF	10.1	<0.05	PVA/H_3PO_4	0-0.8	45.2 @ 0.3 mA cm^{-2}	8.08	0.79	2000/82.2%	[16]
	Carbon onion	7	<0.05	TEABF4/PC	0-3	1.7 @ 1 v/s	140	0.7	10000/NA	[3]
	PANI-Graphene	0.25	<0.001	PVA/H_3PO_4	0-1	3.8 @ 1 mV s^{-1}	46.5	0.575	20000/100%	[17]
	LSG	7.6	0.02	PVA/H_3PO_4	0-1	2.32 @ 1 μA cm^{-2}	38	0.304	2000/97%	[1]
	rGO	0.35	0.01	PVA/H_3PO_4	0-1	0.268 @ 10 mV s^{-1}	0.2	0.02	1000/97%	[18]
	MWCNTs	5	0.01	PVA/H_3PO_4	-0.4-0.4	2.75 @ 10 mV s^{-1}	0.095	0.02	10000/98%	[19]
	MWCNTs	1	0.001	PVA/H_3PO_4	0-0.8	2.02 @ 10 mV s^{-1}	0.0008	0.018	6000/94.1%	[4]
	MnO_2-Au	0.05	0.0005	PVA/H_3PO_4	0-0.8	0.79 @ 10 mV s^{-1}	0.034	0.018	15000/74.1%	[2]
	Graphene	0.045	<0.001	PVA/H_3PO_4	0-1	0.08 @ 10 mV s^{-1}	2.23	0.01	10000/99.1%	[20]
	S-doped graphene	10 nm	NA	PVA/H_2SO_4	0-1	0.582 @ 10 mV s^{-1}	1.191	0.0031	10000/95%	[9]
	rGO	18	0.045	PVA/H_3PO_4	0-1	36.38 @ 10 mV s^{-1}	0.18	0.0026	10000/95%	[6]
	rGO foam	2000	~1	PVA/H_3PO_4	0-1	74.31 @ 5 mV s^{-1}	NA	NA	10000/NA	[21]

	CNTs	27.6	0.12	PVA/H_3PO_4	0-1	4.69 @ 50 mV s^{-1}	NA	NA	2000/93%	[22]

	**Electrode materials**	**Thickness （μm）**	**Aspect radio**	**Electrolyte**	**Voltage (V)**	**Capacity (μAh cm^{-2})**	**Power density (mW cm^{-2})**	**Energy density (uWh cm^{-2})**	**Stability**	**Ref.**
**3D micro battery**	NiSn-LMO	15	0.5	$LiClO_4$/EC/DMC	2-4	NA	0.345	225	15/95%	[23]
	NiSn-LMO	10	0.29	$LiClO_4$/EC/DMC	1.4-3.2	NA	36	65	200/88%	[24]
	Si-NCA	400	3	$LiClO_4$/EC/DMC	3.0-4.3	500 μAh cm^{-2}@1.3C (1800 μAh cm^{-2} @0.12C)	2.11@1.3C (0.64@0.12C)	1600@1.3C (5200@0.12C)	100/92% (7/60%)	[25]
	LFP-LTO	320	11	$LiClO_4$/EC/DMC	1.0-2.5	1500 μAh cm^{-2}@5C	2.7	2694	30/NA	[26]
	LTO-LCO	30	0.5	$LiClO_4$/EC/DMC	2.35	270 μAh cm^{-2}@2C	1.27	634.5	20/60%	[27]
	LMO_1-LMO_2	180	1	$Li_{0.55}La_{0.35}TiO_3$	1.2	32 μAh cm^{-2}	0.0075	38.4	NA	[28]
**Thin film battery**	ThinergyMEC120				3.9	300 μAh/cell	0.18	362.7		
	Enfilm^{TM}(ST)				3.9	700 μAh/cell	0.21	413.33		

Note: NA: data not available. MWCNTs: multiwalled carbon tube. rGO: reduced graphene oxide. LSG: laser scribed graphene oxide. PANI-G: polyaniline on graphene. PANI nF: polyaniline nano fiber.AC: active carbon. LIG-MnO2: MnO2 deposition on laser induced graphene. NiSn-LMO: nickel–tin (anode) and lithium manganese oxide (cathode). LTO-LFP: $Li_4Ti_5O_{12}$ (anode) and $LiFePO_4$ (cathode). LTO-LCO: $Li_4Ti_5O_{12}$ (anode) and LiCoO2 (cathode). LMO_1-LMO_2: $LiMn_2O_4$ and $Li_4Mn_5O_{12}$. $LiClO_4$/EC/DMC: $LiClO_4$ in in ethylene carbonate/diethyl carbonate (1/1, v/v). Si-NCA: Silicon and $LiNi_{0.8}Co_{0.15}Al_{0.05}O_2$. The power density of thin-film batteries was estimated at C/2 rate.

Supplemental reference list

[1] M. F. El-Kady, R. B. Kaner, *Nat. Commun.* 2013, 4, 1475.
[2] W. P. Si, C. L. Yan, Y. Chen, S. Oswald, L. Y. Han, O. G. Schmidt, *Energ. Environ. Sci.* 2013, 6, 3218.
[3] D. Pech, M. Brunet, H. Durou, P. H. Huang, V. Mochalin, Y. Gogotsi, P. L. Taberna, P. Simon, *Nat. Nanotechnol.* 2010, 5, 651.
[4] L. Liu, D. Ye, Y. Yu, L. Liu, Y. Wu, *Carbon* 2017, 111, 121.
[5] S. Wang, B. Hsia, C. Carraro, R. Maboudian, *J. Mater. Chem. A* 2014, 2, 7997.
[6] X. W. Yun, Z. Y. Xiong, L. Tu, L. Q. Bai, X. G. Wang, *Carbon* 2017, 125, 308.
[7] L. Li, J. Zhang, Z. Peng, Y. Li, C. Gao, Y. Ji, R. Ye, N. D. Kim, Q. Zhong, Y. Yang, H. Fei, G. Ruan, J. M. Tour, *Adv. Mater.* 2016, 28, 838.
[8] Z. S. Wu, K. Parvez, S. Li, S. Yang, Z. Liu, S. Liu, X. Feng, K. Müllen, *Adv. Mater.* 2015, 27, 4054.
[9] Z. S. Wu, Y. Z. Tan, S. H. Zheng, S. Wang, K. Parvez, J. Q. Qin, X. Y. Shi, C. L. Sun, X. H. Bao, X. L. Feng, K. Mullen, *J. Am. Chem. Soc.* 2017, 139, 4506.
[10] K. Wang, W. J. Zou, B. G. Quan, A. F. Yu, H. P. Wu, P. Jiang, Z. X. Wei, *Adv. Energy. Mater.* 2011, 1, 1068.
[11] K. Guo, Y. H. Wan, N. Yu, L. T. Hu, T. Y. Zhai, H. Q. Li, *Energy Storage Materials* 2018, 11, 144.
[12] Q. Jiang, N. Kurra, C. Xia, H. N. Alshareef, *Adv. Energy. Mater.* 2017, 7, 1601257.
[13] J. Pu, X. H. Wang, T. Y. Zhang, S. W. Li, J. H. Liu, K. Komvopoulos, *Nanotechnology* 2016, 27, 045701.
[14] Z. S. Wu, K. Parvez, S. Li, S. Yang, Z. Liu, S. Liu, X. Feng, K. Mullen, *Adv. Mater.* 2015, 27, 4054.
[15] D. Zhao, C. Chen, Q. Zhang, W. Chen, S. Liu, Q. Wang, Y. Liu, J. Li, H. Yu, *Adv. Energy. Mater.* 2017, 7, 1700739.
[16] C. Z. Meng, J. Maeng, S. W. M. John, P. P. Irazoqui, *Adv. Energy. Mater.* 2014, 4, 1301269.
[17] J. Ye, H. Tan, S. Wu, K. Ni, F. Pan, J. Liu, Z. Tao, Y. Qu, H. Ji, P. Simon, Y. Zhu, *Adv. Mater.* 2018, 30, 1801384.
[18] W. J. Hyun, E. B. Secor, C.-H. Kim, M. C. Hersam, L. F. Francis, C. D. Frisbie, *Adv. Energy. Mater.* 2017, 7, 1700285.
[19] S. K. Kim, H. J. Koo, A. Lee, P. V. Braun, *Adv. Mater.* 2014, 26, 5108.
[20] Z. S. Wu, K. Parvez, X. L. Feng, K. Mullen, *Nat. Commun.* 2013, 4, 2487.
[21] W. B. Li, Y. H. Li, M. Su, B. X. An, J. Liu, D. Su, L. H. Li, F. Y. Li, Y. L. Song, *J. Mater. Chem. A* 2017, 5, 16281.
[22] W. Yu, H. Zhou, B. Q. Li, S. Ding, *ACS Appl. Mater. Interfaces* 2017, 9, 4597.
[23] J. H. Pikul, H. G. Zhang, J. Cho, P. V. Braun, W. P. King, *Nat. Commun.* 2013, 4, 1732.
[24] H. L. Ning, J. H. Pikul, R. Y. Zhang, X. J. Li, S. Xu, J. J. Wang, J. A. Rogers, W. P. King, P. V. Braun, *Proc. Natl. Acad. Sci. U. S. A.* 2015, 112, 6573.
[25] J. I. Hur, L. C. Smith, B. Dunn, *Joule* 2018, 2, 1187.
[26] K. Sun, T.-S. Wei, B. Y. Ahn, J. Y. Seo, S. J. Dillon, J. A. Lewis, *Adv. Mater.* 2013, 25, 4539.
[27] K. Yoshima, H. Munakata, K. Kanamura, *J. Power Sources* 2012, 208, 404.

[28] M. Kotobuki, Y. Suzuki, H. Munakata, K. Kanamura, Y. Sato, K. Yamamoto, T. Yoshida, *Electrochim. Acta* 2011, 56, 1023.
